# Gene-expression profiling of microdissected breast cancer microvasculature identifies distinct tumor vascular subtypes

**DOI:** 10.1186/bcr3246

**Published:** 2012-08-20

**Authors:** François Pepin, Nicholas Bertos, Julie Laferrière, Svetlana Sadekova, Margarita Souleimanova, Hong Zhao, Greg Finak, Sarkis Meterissian, Michael T Hallett, Morag Park

**Affiliations:** 1 Department of Biochemistry, McGill University, 3655 Promenade Sir William Osler, Montréal, Québec, H3G 1Y6, Canada; 2 McGill Centre for Bioinformatics, McGill University, 3649 Promenade Sir William Osler, Montréal, Québec, H3G 0B1, Canada; 3 Breast Cancer Functional Genomics Group, 1160 Ave. des Pins Ouest, Montréal, Québec, H3A 1A3, Canada; 4 Rosalind and Morris Goodman Cancer Research Centre, McGill University, 1160 Ave. des Pins Ouest, Montréal, Québec, H3A 1A3, Canada; 5 Sequenta, South San Francisco, CA 94080, USA; 6 Merck, Palo Alto, CA 94304, USA; 7 Fred Hutchinson Cancer Research Center, Seattle, WA 98101, USA; 8 Department of Surgery, McGill University and Cedars Breast Clinic, McGill University Health Centre, 687 Ave. des Pins Ouest, Montréal, Québec, H3A 1A1, Canada; 9 Department of Oncology, McGill University and Department of Medicine, McGill University Health Centre, 687 Ave. des Pins Ouest, Montréal, Québec, H3A 1A1, Canada

## Abstract

**Introduction:**

Angiogenesis represents a potential therapeutic target in breast cancer. However, responses to targeted antiangiogenic therapies have been reported to vary among patients. This suggests that the tumor vasculature may be heterogeneous and that an appropriate choice of treatment would require an understanding of these differences.

**Methods:**

To investigate whether and how the breast tumor vasculature varies between individuals, we isolated tumor-associated and matched normal vasculature from 17 breast carcinomas by laser-capture microdissection, and generated gene-expression profiles. Because microvessel density has previously been associated with disease course, tumors with low (*n *= 9) or high (*n *= 8) microvessel density were selected for analysis to maximize heterogeneity for this feature.

**Results:**

We identified differences between tumor and normal vasculature, and we describe two subtypes present within tumor vasculature. These subtypes exhibit distinct gene-expression signatures that reflect features including hallmarks of vessel maturity. Potential therapeutic targets (*MET, ITGAV*, and *PDGFRβ*) are differentially expressed between subtypes. Taking these subtypes into account has allowed us to derive a vascular signature associated with disease outcome.

**Conclusions:**

Our results further support a role for tumor microvasculature in determining disease progression. Overall, this study provides a deeper molecular understanding of the heterogeneity existing within the breast tumor vasculature and opens new avenues toward the improved design and targeting of antiangiogenic therapies.

## Introduction

The growth of tumors beyond a certain size requires the recruitment of an adequate blood supply, which is supplied by abnormal angiogenesis. This involves the triggering of an "angiogenic switch" [1], whereby the tumor microenvironment enters a proangiogenic mode in response to hypoxia. This process is accompanied by increased levels of multiple proangiogenic factors, including vascular endothelial growth factor A (VEGFA) and platelet-derived growth factor B (PDGFB), as well as decreases in antiangiogenic factors such as endostatin. This leads to an increase in the number of proliferating endothelial cells, along with enhanced endothelial cell recruitment and migration toward the tumor bed [2].

Because endothelial cells are considered to be genetically stable [2], modulation of angiogenic processes is considered to be a promising area for cancer therapy. However, clinical trials of antiangiogenic therapies in breast cancer have reported mixed results [3-5]. Heterogeneity among tumor vasculatures may partially explain this lack of consistency [3]. The most common measure of vascular heterogeneity is microvessel density (MVD), which has weak prognostic value in breast cancer [6]. MVD is significantly associated with histologic type, but does not appear to be linked to other features, including nodal status, tumor grade, estrogen receptor (ER) status, molecular subtype, or the presence of vascular invasion [7,8]. However, other characteristics of the tumor vasculature, such as vascular proliferation and levels of circulating endothelial progenitor cells, also have clinical significance in cancer [9,10]. Therefore, a more complete understanding of vascular heterogeneity is required.

Gene-expression profiling studies have provided a greater understanding of breast tumor heterogeneity [11,12] and have highlighted the role of the stroma in influencing disease outcome [13-16]. However, previous studies of tumor vasculature have mostly been limited to small numbers of samples and have focused on direct comparisons between tumor-associated and normal vasculature [17-23]. Although these studies have identified alterations characteristic of the tumor vasculature *per se*, they have not addressed the question of vascular heterogeneity or investigated whether distinct intertumoral vascular subtypes exist. Here, we use laser-capture microdissection (LCM) to isolate tumor-associated and matched normal microvascular compartments separately from 17 human breast cancer samples. After gene-expression profiling, we identified and validated gene-expression signatures that define two distinct subtypes of tumor vasculature, an understanding of which would represent an important step toward the improved design and targeting of therapeutic modalities.

## Materials and methods

Tissue samples from 21 patients undergoing surgery for primary invasive ductal carcinoma (IDC) with no prior neoadjuvant therapy, and from one patient undergoing reduction mammoplasty, were subjected to LCM (see Additional File 1, Table S1A, B). From this cohort, we obtained 17 samples of tumor-associated vasculature; in 15 cases, we also obtained normal vasculature. After determination of MVD (see Additional File 2, Figure S1A), PECAM1-positive cells (microvasculature) were collected by using LCM from within tumor beds (*n *= 17), as well as from morphologically normal tissue adjacent to breast tumors (*n *= 14) and from one reduction mammoplasty (sample V) (see Additional File 2, Figure S1B). Only PECAM1-positive cells present within capillaries and small vascular structures were collected, avoiding isolated PECAM1-positive cells and vessels with large lumens. Total RNA was extracted, amplified, labeled, and hybridized on Agilent 44K Whole Genome arrays (Agilent Technologies, Santa Clara, CA). The endothelial and epithelial cell content of isolated samples was assessed by quantitative real-time PCR (qRT-PCR) (see Additional File 2, Figure S1C).

### Description of samples

Clinical information was collected on a prospective basis (median follow-up, 5.6 years; see Additional File 1, Table S1B). Occurrence of distant recurrence was determined by examination of medical records. Matched normal epithelial samples were obtained as previously described [24]. This study was approved by the McGill University Health Centre Research Ethics Board (Protocols SDR-99-780 and SDR-00-966). All patients provided written, informed consent.

### Laser-capture microdissection, RNA isolation, and microarray hybridization

Immunohistochemistry directed against PECAM1 for MVD quantitation was carried out as per the antibody manufacturer's instructions. Quantitation of PECAM1 staining density was performed by averaging the stained pixel intensity of three fields captured at 10 × magnification by using imageJ [25]. Details of the anti-PECAM1 immunohistochemistry protocol used for guided LCM are presented in Supplementary Information (see Additional File 3, Supplementary Materials and Methods). LCM, RNA isolation and sample preparation, as well as microarray hybridization, were carried out as previously described [13]. Microarray results have been submitted to the Gene Expression Omnibus (GSE15363).

### Differential expression, class discovery, and class prediction

All analyses were performed using R/Bioconductor version 2.6 [26]. Microarray data were extracted as described in Supplementary Information (see Additional File 3, Supplementary Materials and Methods). Initially, the set of most-variable probes (*n *= 1,168) was defined as those with an interquartile range above 2. Class discovery was performed by hierarchical clustering based on this set, using Ward's minimum-variance method with a correlation distance metric. The significance of the resulting clusters was calculated by using the pvclust package with 10,000 bootstrap iterations. Heatmaps were generated by scaling each row (gene) into Z-scores, subtracting the mean and dividing by its standard deviation. Differential expression between sample sets was determined by using Linear Models for Microarray Analysis (LIMMA) [27]. For genes represented by multiple probes, only the probe with the largest interquartile range was used. Unless stated otherwise, a gene was considered differentially expressed if the FDR-adjusted *P *value was ≤0.05 [28].

Predictors of recurrence were generated as follows: gene expression for subtype B member profiles was adjusted by removing the average differences between subtype B and subtype A tumor vascular expression for each gene. Then, the six most significantly differentially expressed genes between recurrent and nonrecurrent tumor vasculature were identified by using a Wilcoxon rank-sum test. These genes were used to train a linear discriminant analysis classifier and tested for accuracy through leave-one-out cross-validation (LOOCV). All steps (including the adjustment of tumor vascular subtypes) were done under cross-validation. Gene sets of size two to 100 were tested for prediction, and six-gene predictors were found to exhibit the highest accuracy under LOOCV.

### Quantitative real-time PCR and immunohistochemistry

Validation for selected genes and cognate proteins was performed by qRT-PCR and IHC, respectively, on subsets of samples. Full details and probe sequences are presented in Supplementary Information (see Additional File 3, Supplementary Materials and Methods).

## Results and discussion

Because our goal was to investigate the heterogeneity of the tumor microvasculature in invasive ductal carcinomas, samples with high and low MVD were chosen to maximize the spectrum of heterogeneity present within the sample cohort. Within this set, we identified tumor samples with high (*n *= 8) or low (*n *= 9) MVD, as determined by immunohistochemistry directed against the endothelial marker PECAM1 (see Additional File 1; Table S1B; Additional File 2, Figure S1A).

### Isolation of vasculature-enriched samples and gene-expression profiling

A significant enrichment of *PECAM1 *was observed in microdissected vasculature when compared with the epithelial cell marker cytokeratin 8 (*KRT8*); conversely, the latter marker was enriched in matched microdissected epithelial RNA samples (see Additional File 2, Figure S1C). These results indicate that the vascular samples are highly enriched in endothelial cell content.

### Tumor and normal vasculatures possess distinct expression profiles

**Figure 1:**
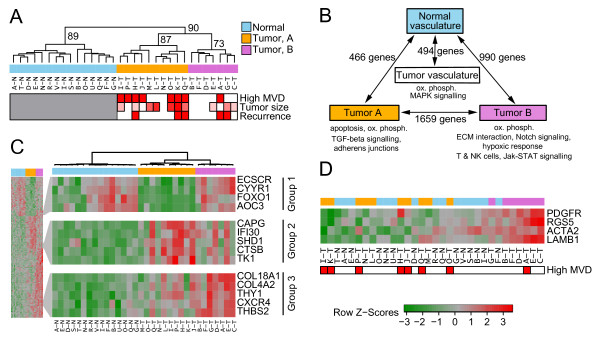
**Gene expression identifies two distinct subtypes of breast tumor vasculature**. **(A) **Hierarchical clustering separates normal (blue) and tumor vascular samples and segregates the tumors into two clusters (orange, magenta). Clusters in the tree are labeled with the percentage of times they were observed in 10,000 bootstrap iterations. Clinical characteristics for tumor samples: red, high/positive; white, low/negative. Tumor size is represented as a gradient from white (smallest tumor) to red (largest tumor). Clinical variables are not presented for samples from normal vasculature (left side). **(B) **Schematic depiction of pathways and processes differentially expressed between normal vasculature, tumor vasculature as a whole, and the two tumor vascular subtypes. Edges are labeled with the number of differentially expressed genes for each comparison. **(C) **Heatmap of genes differentially expressed between any two of the normal, A and B subtypes (FDR < 0.0001). Also presented are details of three functionally related groups: 1, normal vascular genes; 2, angiogenesis-inhibitor genes; and 3, classic tumor vascular genes. Heatmap colors represent Z-scores for each gene (red, high expression; green, low expression). **(D) **Ordering of samples by expression of pericyte markers demonstrates that these are more highly expressed in subtype B members.

To investigate the differences among the expression profiles generated, we performed hierarchical clustering by using the 1,168 most variable genes (interquartile range >2) over the entire dataset. The normal and tumor vascular samples formed two distinct clusters (Figure 1A; see Additional File 2, Figure S2A). Further comparison of the tumor and normal vasculature samples identified 494 differentially expressed genes (FDR < 0.05, LIMMA; see Additional File 4, Sheet 1).

A significant overlap exists between the gene sets discriminating between tumor and normal vasculature identified by previous studies [17-23] and here (*P *< 0.05 for each, Fisher Exact test; Figure S3A). This overlap is significant even for the studies performed on different tumor types [19-23] or by using other isolation techniques [18-20,23]. Common processes include extracellular matrix remodeling and cytokine signaling [29] (see Additional File 1, Table S2). Several genes previously reported to be elevated in tumor vasculature [29] are also elevated in our tumor vascular cohort, including osteonectin (*SPARC*), collagens 1A1, 3A1, 4A2, and 18A1 (*COL1A1, COL3A1, COL4A2 and COL18A1*), and Thy cell-surface antigen (*THY1*) (see Additional File 4, Sheet 1). This is consistent with the observation that tumor vasculature is constantly reorganizing [30,31] and confirms that our normal and tumor vasculature are comparable to those isolated in previous studies.

The tumor vasculature also exhibits differential expression of genes related to oxidative phosphorylation/stress and MAPK signaling (Figure 1B; see Additional File 1, Table S2; Additional File 4, Sheet 1). It is known that reoxygenation of vasculature, consistent with the variable flow of blood in tumor vessels, leads to the creation of reactive oxygen species [32].

### Tumor vascular expression profiles segregate into two distinct subtypes reflecting different biological processes

Interestingly, the hierarchical clustering revealed that the tumor samples form two distinct clusters, here termed A and B (Figure 1A; see Additional File 1, Figure S2A); this separation was also reflected in the first component when the set of tumor vascular samples were subjected to principal-component analysis (see Additional File 1, Figure S2B). Analysis of differential expression between these two clusters identifies 1,659 genes (FDR < 0.05, LIMMA; see Additional File 4, Sheet 2). This is more than 3 times larger than the number of genes differentially expressed between normal and tumor samples, emphasizing the significance of the clusters and indicating that homogeneity within each cluster is higher than that within the tumor vasculature as a whole. Thus, separating tumor samples by cluster may prove more informative than grouping them together.

The tumor vascular clusters are independent of major clinical variables, including ER and HER2 status, grade, and lymph node involvement (*P *≥ 0.6; see Additional File 1, Table S1A) but are enriched for larger and smaller tumors as well as high and low MVD (*P <*0.05, *t *test). Interestingly, no statistically significant differential gene expression was found between high- and low-MVD samples (FDR < 0.05, LIMMA). This demonstrates that although MVD status is associated with the tumor vascular clusters identified here, additional properties of the samples are primarily responsible for the molecular profiles and observed clustering.

To identify biological functions specifically associated with each of the two subtypes of tumor vasculature, we identified the most highly differentially expressed genes (FDR < 0.0001, LIMMA, Figure 1C; FDR < 0.05, LIMMA; see Additional File 4, Sheet 2) and pathways (FDR < 0.05; Figure 1B; see Additional File 1, Table S2) between normal vasculature and each of the tumor vascular subtypes A and B.

Genes within Group 1 exhibit the highest expression in normal samples and the lowest expression in tumor-associated vasculature subtype A members (Figure 1C). Interestingly, these include *AOC3 *and *FOXO1*, both associated with vascular shear stress [33,34]. Reduced shear stress suggests a decreased flow rate, consistent with the hypothesis that tumor vessels, and in particular those belonging to subtype A, lack appropriate perfusion [35]. Many genes and processes related to metabolism and biosynthesis are also overrepresented in subtype A (see Additional File 1, Table S2; Additional File 4, Sheet 2), suggesting active proliferation. Consistent with this, Group 2, consisting of genes whose expression is elevated in subtype A (Figure 1C), includes cathepsin B (*CTSB*), associated with active vascular remodeling [35,36].

Genes within Group 3 are more highly expressed in subtype B, and are enriched for elements involved in interactions with the extracellular matrix, collagen production, focal adhesion, hypoxia, glycolysis, immune response, and protein export, suggesting that this group may represent a more stable vasculature (Figure 1C; Additional File 1, Table S2). These include genes involved in antiangiogenic processes, such as thrombospondin 2 (*THBS2*) and the collagens *COL18A1 *and *COL4A2*, which can be cleaved to form endostatin and canstatin, respectively [37]. Antiangiogenic signaling from these and other genes (see Additional File 1, Figure S3B) may contribute to the decreased overall MVD seen in these samples. Interestingly, *NOTCH3 *is also elevated in subtype B (see Additional File 4, Sheet 2); Notch signaling is implicated in vascular development and maturation [38,39].

**Figure 2:**
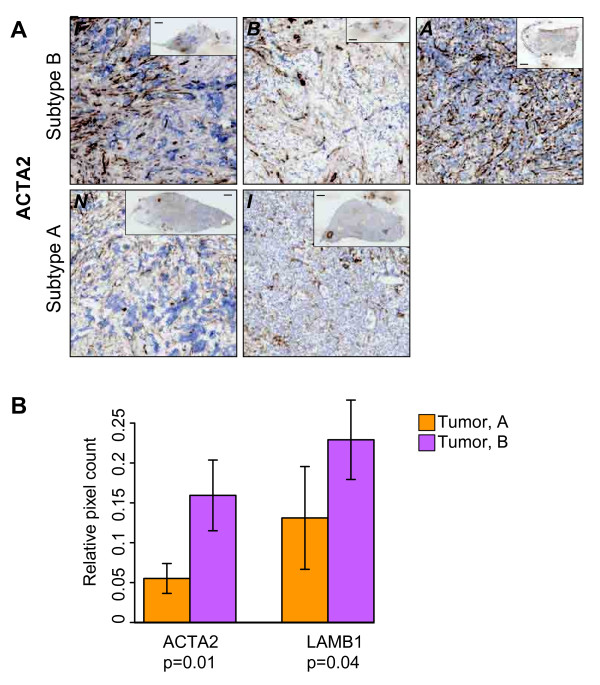
**Immunohistochemistry directed against selected subtype markers confirms their differential expression between subtypes**. **(A) **Anti-ACTA2 immunohistochemistry. Individual sample labels are indicated in italic font in the upper left corner of each image. Each image is of a representative 1 × 1-mm area; inset depicts whole section; scale bar in inset, 1 mm. **(B) **Bar plot of pixel counts for IHC staining of ACTA2 and LAMB1, relative to PECAM1 staining.

Tumor vasculature is often considered immature with respect to normal vasculature [30], the known functions of many of the individual genes identified here suggest that the two tumor vascular clusters may represent differences in degree of vascular maturity. This hypothesis was investigated by using several approaches. Pericytes line mature capillary vessels and would be co-isolated with PECAM1-positive endothelial cells during LCM, whereas the association of pericytes with immature vessels is relatively loose, and this cell type would therefore be co-isolated at a lower frequency in such vessels [31]. Therefore, we explored whether clusters A and B differed with respect to their content of pericyte-specific signals, specifically the pericyte markers *ACTA2, PDGFRβ*, and *RGS5 *[40], as well as *LAMB1*, a surrogate of laminin-8 (α4β1γ1) implicated in vessel maturation [41] (Figure 1D). Interestingly, these markers are elevated in cluster B (*P <*0.05 for each; *t *test), and their expression pattern successfully recapitulates the clusters (Figure 1D). Differential expression of *PDGFR*β was confirmed by qRT-PCR on amplified RNA prepared from tumor vascular samples (see Additional File 2, Figure S3C), whereas immunohistochemistry (IHC)-based validation was also performed for ACTA2 and LAMB1 (Figure 2A, B; see Additional File 2, Figure S4A). Serial staining directed against PECAM1 showed that most ACTA2 and LAMB1 expression is observed in proximity to the PECAM1-expressing vasculature (see Additional File 2, Figure S4B, C), while pixel counts over whole sections (normalized for PECAM1 staining, as assessed on a neighboring section) showed a significantly increased expression of ACTA2 and LAMB1 in tumors belonging to cluster B (*P <*0.05 for each, Wilcoxon test; Figure 2C; Additional File 1, Table S1A). Therefore, the differential gene expression observed between clusters is recapitulated at the protein level for these markers. In addition, assessment of *LAMB1 *mRNA content in selected samples by qRT-PCR significantly correlated with the microarray-derived values; this also held true for several other selected genes (see Additional File 2, Figure S5). Supporting this observation, a negative correlation between MVD and pericyte coverage has also been identified in endometrial cancer [9].

To assess whether the subtype B-specific gene-expression profile is driven by pericyte-specific genes, the sample orderings induced by pericyte marker expression and subtype-specific gene expression were compared (see Additional File 2, Figure S6). First, we confirmed that ordering of all samples by relative expression of known pericyte markers recapitulated the separation of subtype A and B members (Additional File 2, Figure S6A, B); this ordering was not correlated with expression of endothelial marker genes (Additional File 2, Figure S6C). Subtype B members were then ordered by relative expression of known pericyte markers (Additional File 2, Figure S6D). Then, retaining this ordering, the relative expression of the top 200 genes differentially expressed between the A and B subtypes (ranked by *P *value) was examined (see Additional File 2, Figure S6E). Importantly, no correlation was seen at the sample level between relative pericyte-marker content and relative expression levels of subtype-specific genes within the set of subtype B members, supporting that processes other than those linked to pericyte-marker content distinguish these two subtypes.

Pathway analysis highlights an increased expression of genes related to glycolysis and oxidative phosphorylation in subtype B (Figure 1B). This is intriguing, as elevation of either glycolysis or oxidative phosphorylation often leads to downregulation of the other [42]. Coupled with an increased expression of ribosomal genes, the increase in oxidative phosphorylation could be evidence of increased metabolism in subtype B vasculature. Interestingly, the different tumor vascular subtypes also vary at the level of NK and T-cell marker expression, suggesting that vascular subtypes can affect the tumor microenvironment. Additionally, *CXCL12/SDF-1*, considered to play a role in pericyte recruitment [43], is decreased in subtype A tumor vasculature relative to subtype B, consistent with the presence of increased levels of pericyte markers in subtype B, whereas angiopoietin-2 (*ANGPT2*), thought to promote blood-vessel destabilization by acting as an ANGPT1 antagonist [44], is elevated in subtype B tumor vasculature.

Together, these results strongly support the hypothesis that the vascular clusters observed here reflect differences in vessel maturity, and indicate that related processes, including anti- and proangiogenic regulators and matrix remodeling, also contribute to the segregation of the vascular subtypes. We suggest that cluster B, associated with low MVD, may represent samples with a more mature tumor vasculature with respect to members of cluster A. Other potential factors driving cluster segregation may include unidirectional shear stress (USS), expected to be highest in vessels of subtype B. USS elicits an antiinflammatory response [45] and has been shown to induce miR-21 expression in endothelial cells, leading to decreased apoptosis and activation of the nitric oxide pathway, implicated in Notch signaling [46]. All of these are consistent with our proposed subtypes (Figure 1B). Interestingly, genes previously identified as being commonly overexpressed in tumor endothelium and therefore proposed as specific markers for endothelial cell targeting, including *SPARC *and *COL4A1 *[47], are elevated in subtype B but not subtype A tumor vasculature, accentuating the importance of gaining a better understanding of the heterogeneity present in this compartment.

### Most previous signatures of tumor vascular cells identify only tumor vascular subtype B

**Figure 3:**
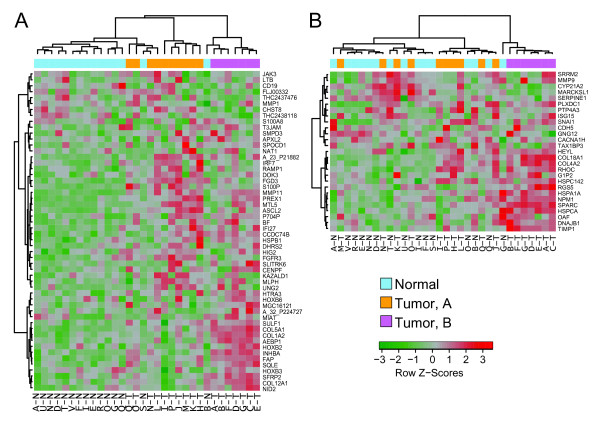
**Tumor context and tissue of origin affects vascular expression in tumor samples**. **(A) **The tumor vascular signature from Bhati *et al. *[17] separates tumor from normal vasculature in our samples, and also segregates most members of the A and B subtypes. **(B) **The tumor vascular signature from Parker *et al. *[18] identifies subtype B tumor vasculature, but fails to segregate subtype A tumor vasculature from normal samples.

Previous studies (see Additional File 2, Figure S3A) have generated signatures that segregate tumor versus normal vascular samples, but have not identified distinct subtypes within the tumor vasculature. To test whether these signatures can differentiate between our tumor vascular subtypes, we examined the clustering induced by previously reported tumor vascular signatures [17,18,21-23] in our dataset. All previously published tumor vascular gene sets investigated could segregate members of subtype B from normal samples in our data (Figure 3; Additional File 2, Figure S3D-G); however, only the gene set described by Bhati *et al. *[17] generated from breast tumors using LCM, successfully segregates the subtype A and normal samples (Figure 3A). The inability of most existing datasets to generate signatures capable of distinguishing between tumor vascular subtypes may be partially explained by differences in the isolation technique used. Unlike LCM, immunomagnetic bead-based approaches using dispersed cells fail to isolate cells adjacent to the vessels that can provide additional information about the vascular microenvironment. Additionally, differences in the tissue of origin may contribute to these observations (see Additional File 2, Figure S3A), suggesting that subtype A may be enriched in breast tumors, and supporting the concept of tissue-specific antiangiogenic approaches.

The ability of the tumor vascular signature from Bhati *et al. *[17] to partially recapitulate our tumor vascular subtypes (Figure 3A) suggests that their samples contained features of both subtypes, and indicates that this vascular heterogeneity is likely to be common in breast cancer. Bhati *et al. *[17] used laser-capture microdissection to isolate factor VIII-expressing vascular cells from human breast cancers and normal breast tissue; however, they failed to identify subtypes within the tumor sample set. This may partly be due to the restricted sample size used in their study (*n *= 5), whereas the increased size of our patient cohort (n = 17), in combination with our selection based on MVD, likely enabled us to capture a broader range of interpatient variation in tumor vasculature. The small number of samples investigated in most previous studies (n = 1 to 5) [17-20,22,23] also raises the possibility that only members of one subtype were present in their study cohorts. Hence our identification and initial characterization of distinct breast cancer vasculature subtypes is a novel finding that emphasizes the concept that breast cancer heterogeneity extends to the different subcomponents of the tumor microenvironment.

### Gene-expression differences induced by subtype membership mask differential gene expression associated with disease outcome

**Figure 4:**
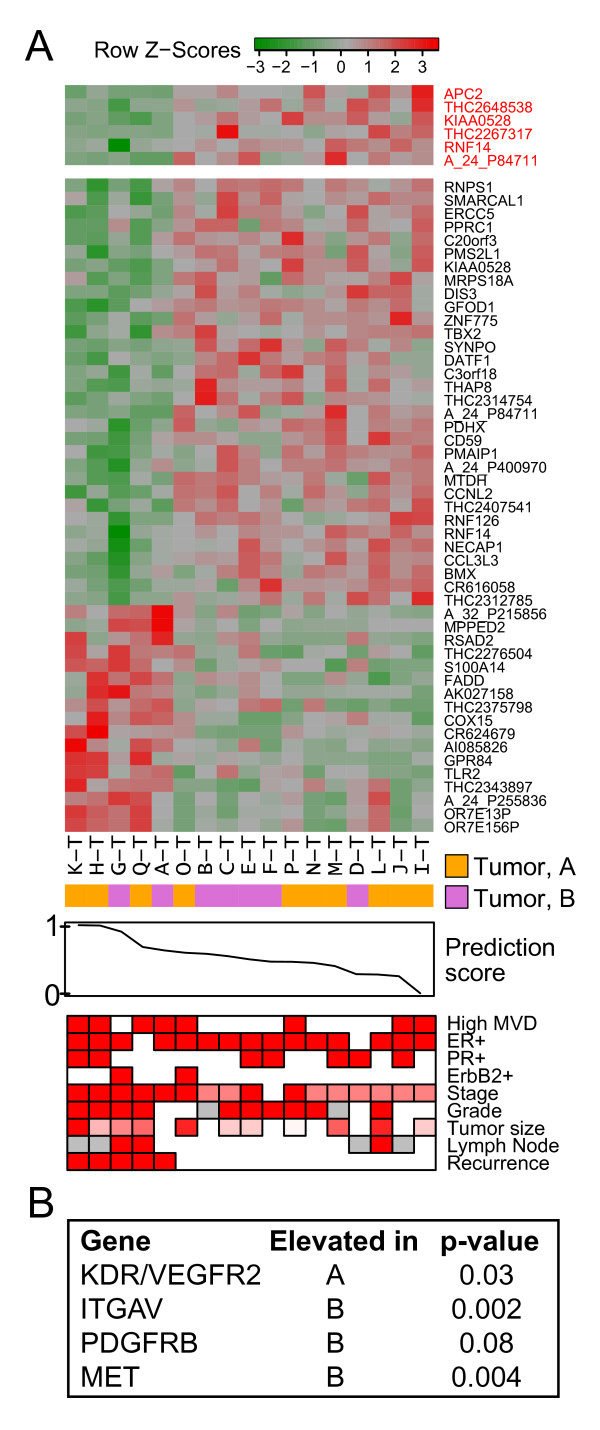
**Tumor vascular subtypes suggest potential prognostic and predictive biomarkers**. **(A) **Heatmap depicting expression of the six genes composing the prognostic predictor (above, in red), as well as the top 50 (Table S3) genes differentially expressed with recurrence in tumor vascular samples. A higher prediction score represents a higher likelihood of recurrence. The prediction score was calculated under cross-validation. Clinical variables are presented as in Figure 1A. **(B) **List of antiangiogenic drug targets [56] whose expression differs between the tumor vascular subtypes.

To determine whether differences in tumor vasculature gene expression were correlated with other aspects of tumor biology and disease course, we performed differential expression analysis between the five recurrent and 12 nonrecurrent tumor samples in our dataset. This identified a total of 128 genes (*P *< 0.01, no adjustment: see Additional File 1, Table S3), including *TLR2 *(involved in innate immunity) [48] and *GAB1 *(a scaffold protein linked to VEGF signaling and endothelial migration) [49]. Subtype membership is not correlated with recurrence in our relatively small dataset. This agrees with previous reports that differences in vessel maturity are associated with lymph node status but not other clinical variables in breast cancer [50]. However, we speculated that the differences in gene expression induced by subtype membership might be sufficiently large to mask differential gene expression associated with disease outcome. Similar conclusions have been drawn regarding gene expression-based predictors of breast cancer outcome based on whole-tumor data, which have generally been found to be more reliable when limited to specific tumor subtypes [51,52]. Generation of subtype-specific predictors was not possible, given the small size of each group and the limited number of recurrences in each (see Additional File 1, Table S1A,B). Therefore, we investigated this hypothesis by removing the gene-expression changes that could be ascribed to subtype membership. This was achieved by calculating the difference in average expression between the members of the two subtypes for each probe, and subtracting this value from the relevant probe data for all members of one subtype. After this adjustment, we could successfully build a six-gene predictor that identified recurrent patient samples with 94% accuracy under cross-validation in our dataset (*P *= 0.002; Fisher Exact test; Figure 4A). Without applying this correction, no successful predictor could be constructed. The fact that the combination of these genes can predict recurrence under cross-validation within our dataset confirms their differential expression, despite their individual lack of significance after FDR adjustment.

This indicates that vascular markers of recurrence can be subtype-specific and that the subtypes contain different markers linked to recurrence. Additionally, the observation that successful construction of a predictor cannot be achieved without taking the inter-subtype differences into account suggests that these differences are sufficiently large to mask signals associated with other clinical variables, and supports that tumor vascular subtypes must be taken into account in future analyses of this compartment.

Our earlier work established that breast tumor stroma as a whole contains sufficient information to predict recurrence independently [13]. This study highlights that features of patient-specific vasculatures can reflect differences in the tumor microenvironment predictive of recurrence. As such, the tumor vasculature, including its subtypes, should be considered when studying the determinants of recurrence. This adds another dimension to previous reports that different characteristics of vasculature are associated with tumor stage and metastasis [6,9,10,53], and indicates that the effects of tumor vasculature on disease progression are complex. Further research will be necessary to identify successfully all relevant factors contributed by the vascular environment in tumor progression.

### Drug targets are differentially expressed between subtypes

The existence of vascular subtypes has important implications for the choice of antiangiogenic treatment strategies. A substantial proportion of patients in such clinical trials fail to show clinical benefits [3,4]; short-term treatment with one antiangiogenic drug led to an increased incidence of metastasis [54]. Importantly, observed differences between our subtypes (for example, pericyte coverage) may reflect mechanisms of resistance to antiangiogenic therapy [4]. Pericyte coverage can reduce tumor vessel vulnerability to VEGF inhibition, and in such cases, targeting treatments against both pericytes and endothelial cells may lead to improved efficacy [55].

The targets of several antiangiogenic drugs [56,57] are also differentially expressed between subtypes (Figure 4B). Both *PDGFRβ *and *MET *(a receptor tyrosine kinase, whose natural ligand HGF is a potent angiogenic factor [58]), are elevated in subtype B; antagonists to α_v _integrin (*ITGAV*) [59], also elevated in subtype B, have been shown to block neovascularization. Vessel maturity has been linked to resistance to antiangiogenic therapy in melanoma [60], suggesting that this mechanism may be applicable across tumor types. The differential expression of drug targets indicates that subtype-specific approaches to targeting tumor angiogenesis may lead to improved response rates by permitting prestratification of patient populations. Modifications of the tumor vasculature, as suggested by a recent study identifying vessel normalization through ablation of the endothelial oxygen sensor *PHD2 *as a potential means to improve the delivery and efficacy of chemotherapeutic agents [61], may also have subtype-specific effects. The identification of biomarkers for prediction of response to antiangiogenic therapy is a current critical clinical need [62], and the vascular-subtype gene-expression profiles presented here provide new perspectives for the validation of such markers.

## Conclusions

Overall, our data illustrate the heterogeneity of the breast tumor vasculature, define two tumor vascular subtypes that are associated with, but not defined by, microvessel density, and represent a first step toward establishing the landscape of vascular subtypes in breast cancer. Our previous work has shown that heterogeneity within the stromal compartment of breast tumors is linked to disease outcome [13]. We now suggest that breast tumor vasculature heterogeneity also plays a role in determining disease course, and that further study thereof may be critical for improved patient stratification before selection of specific targeted therapeutic regimens.

## Abbreviations

ER: estrogen receptor; FDR: false discovery rate; HER2: human epidermal growth factor receptor 2; IHC: immunohistochemistry; KEGG: Kyoto Encyclopedia of Genes and Genomes; LCM: laser-capture microdissection; MVD: microvessel density; qRT-PCR: quantitative real-time polymerase chain reaction.

## Competing interests

The authors declare that they have no competing interests.

## Authors' contributions

FP participated in the design and coordination of the study, conducted bioinformatics analyses, and drafted the manuscript. NB supervised the later stages of the experimental work and helped to draft the manuscript. JL contributed to the later stages of the experimental work and helped to draft the manuscript. SS co-conceived of the study and supervised the initial phases of the experimental work. MS conducted immunohistochemistry and laser-capture microdissection experiments. HZ conducted RNA isolation, amplification, microarray, and qRT-PCR experiments. GF assisted with bioinformatics analyses and helped to draft the manuscript. SM provided clinical insights and assisted with obtaining samples. MTH co-supervised the design and coordination of the study, supervised bioinformatics analyses, and helped to draft the manuscript. MP conceived of the study, supervised its design and coordination, and helped to draft the manuscript. All authors read and approved the final manuscript.

## Supplementary Material

Additional file 1 **Supplementary Tables S1 through S3**. **Supplementary Table S1A, B: **Patient information. **Supplementary Table S2**. KEGG pathways differentially expressed between different categories of vasculature. **Supplementary Table S3**. List of genes differentially expressed between samples from recurrent and nonrecurrent cancer patients. Click here for file

Additional file 2 **Supplementary Figures S1 through S6. Supplementary Figure S1 **Specific enrichment of the vasculature by laser capture microdissection. **Supplementary Figure S2 **Heatmap depicting differential gene expression after class discovery within the set of all microdissected endothelial samples and principal component analysis (PCA) of tumor endothelial samples. **Supplementary Figure S3 **Characteristics of the studies in which other tumor vascular signatures were generated, heatmaps representing expression of antiangiogenic genes and the pericyte marker PDGFRβ in tumor vasculature, and heatmaps of clustering induced by other tumor vascular signatures in the current dataset. **Supplementary Figure S4 **Images of anti-ACTA2 IHC, as well as of anti-ACTA2/LAMB1 and anti-PECAM1 IHC carried out on consecutive sections. **Supplementary Figure S5 **qRT-PCR validation for selected genes identified from analyses of microarray data. **Supplementary Figure S6 **Analyses of correlation between cell-type marker content and expression of genes differentially expressed between the A and B subtypes. Click here for file

Additional file 3 **Supplementary Materials and Methods**. Supplementary Materials and Methods with corresponding references. Click here for file

Additional file 4 **Supplementary Datasets Sheets 1 and 2. Sheet 1: **List of genes differentially expressed between normal and tumor vasculature. **Sheet 2A-C: **Lists of genes differentially expressed between normal vasculature and A and B tumor vascular-subtype members. Click here for file
