# Supplementary material for: Gene-expression profiling of microdissected breast cancer microvasculature identifies distinct tumor vascular subtypes
Source: Breast Cancer Res. 2012 Aug 20;14(4):R120. doi: 10.1186/bcr3246 (PMC3680943; doi:10.1186/bcr3246)
Supplement: Additional file 1 — Supplementary Tables S1 through S3. Supplementary Table S1A, B: Patient information. Supplementary Table S2. KEGG pathways differentially expressed between different categories of vasculature. Supplementary Table S3. List of genes differentially expressed between samples from recurrent and nonrecurrent cancer patients. [file bcr3246-S1.DOC]

**Additional File 1 - Supplementary Tables S1-S3**

**Supplementary Table 1. Patient Information.** **A.** Summary of the clinical characteristics of the patients in this study, along with associations with tumor vascular subtype and recurrence. OR, odds ratio of a patient belonging to the B subtype or experiencing recurrence. P, significance of the odds ratio using a t-test or Fisher’s exact test, as appropriate. **B.** Clinical characteristics of individual patients included in the study. The time of follow-up is either the time to recurrence or the time to last follow-up. Pt., patient; LN, lymph node; Rec., recurrence; F/U, follow-up; SMA, SMA-positive pixels as % PECAM1-positive pixels; LAMB1, LAMB1- positive pixels as % PECAM1-positive pixels; NA, data not available/not applicable.

**A.**

| **Characteristic** | **Number** | **Subtype**  **OR** | **Subtype**  **P** | **Recurrence**  **OR** | **Recurrence**  **P** |
| --- | --- | --- | --- | --- | --- |
| **Age (mean +/- SD)** | 59 +/- 8.3 |  | 0.38 |  | 0.069 |
| **Tumor size (mm) (mean +/- SD)** | 22 +/- 8.1 |  | 0.038 |  | 0.29 |
| **Tumor PECAM1 staining density** |  | 12 | 0.05 | 7 | 0.13 |
| high | 8 |  |  |  |  |
| low | 9 |  |  |  |  |
| **ER status** |  | 0.68 | 1 | 2.6 | 0.51 |
| **-** | 2 |  |  |  |  |
| **+** | 15 |  |  |  |  |
| **PR status** |  | 1.1 | 1 | 1.1 | 1 |
| **-** | 10 |  |  |  |  |
| **+** | 7 |  |  |  |  |
| **Her2 status** |  | 1.5 | 1 | 0.39 | 0.51 |
| **-** | 15 |  |  |  |  |
| **+** | 2 |  |  |  |  |
| **Grade** |  |  | 0.6 |  | 0.018 |
| I | 1 |  |  |  |  |
| II | 8 |  |  |  |  |
| III | 8 |  |  |  |  |
| **LN status** |  | 1 | 1 | 0.4 | 0.6 |
| **-** | 5 |  |  |  |  |
| **+** | 10 |  |  |  |  |
| NA | 2 |  |  |  |  |
| **Recurrence** |  | 1.1 | 1 |  |  |
| Yes | 5 |  |  |  |  |
| No | 12 |  |  |  |  |

B.

| **Pt.** | **Age** | **Tumor size (mm)** | **MVD** | **ER/PR/**  **HER2** | **Grade** | **LN** | **Rec.** | **F/U (yrs)** | **SMA** | **LAMB1** |
| --- | --- | --- | --- | --- | --- | --- | --- | --- | --- | --- |
| **A** | 53 | 15 | high | +/-/- | III | - | Yes | 4.67 | 0.18 | 0.2 |
| **B** | 57 | 15 | low | +/-/- | II | NA | No | 1.92 | 0.1 | NA |
| **C** | 69 | 20 | low | +/-/- | II | + | No | 6.34 | 0.21 | 0.3 |
| **D** | 58 | 15 | low | -/+/- | II | - | No | 6.17 | NA | 0.21 |
| **E** | 58 | 20 | low | +/+/- | III | + | No | 5.83 | NA | 0.2 |
| **F** | 55 | 15 | low | +/+/- | I | + | No | 4.75 | 0.15 | NA |
| **G** | 51 | 26 | low | +/-/+ | III | + | Yes | 2.17 | NA | NA |
| **H** | 40 | 22 | high | +/+/- | III | + | Yes | 3.42 | 0.052 | NA |
| **I** | 72 | 20 | high | +/-/- | II | - | No | 7 | 0.038 | 0.19 |
| **J** | 71 | 15 | high | +/+/- | II | - | No | 6.5 | NA | NA |
| **K** | 64 | 38 | high | +/+/- | III | + | Yes | 5.08 | 0.046 | 0.047 |
| **L** | 60 | 35 | low | +/-/- | II | + | No | 6.08 | NA | 0.09 |
| **M** | 70 | 30 | low | +/+/- | II | NA | No | 6.08 | NA | NA |
| **N** | 56 | 15 | low | +/-/- | II | + | No | 5.58 | 0.075 | NA |
| **O** | 55 | 35 | high | +/-/+ | III | - | No | 5.49 | NA | NA |
| **P** | 64 | 16 | high | +/-/- | III | + | No | 5.83 | NA | 0.13 |
| **Q** | 55 | 29 | high | -/-/- | III | + | Yes | 1 | NA | NA |
| **R** | 34 | 8 | NA | -/-/+ | III | NA | Yes | 1.33 | NA | NA |
| **S** | 44 | 17 | NA | +/+/- | II | - | No | 7.75 | NA | NA |
| **T** | 82 | 90 | NA | -/-/- | II | - | No | 5.24 | NA | NA |
| **U** | 46 | 40 | NA | -/-/- | II | + | No | 5.08 | NA | NA |
| **V** | 56 | NA | NA | NA | NA | NA | NA | NA | NA | NA |

**Supplementary Table S2. KEGG pathways differentially expressed between different categories of vasculature.** The “comparison” column represents the two types of vasculature (normal, tumor, A and B) compared; the “elevated in” column denotes which type has elevated expression of genes associated with a given pathway. Pathway ID, KEGG pathway ID number.

| **Pathway name** | **Comparison** | **Elevated in** | **z-score** | **P-value** | **Pathway ID** |
| --- | --- | --- | --- | --- | --- |
| **Oxidative phosphorylation** | Normal vs. tumor | Tumor | -14.20 | 1.61E-43 | 190 |
| **Neuroactive ligand-receptor interaction** | Normal vs. tumor | Normal | 5.17 | 2.15E-05 | 4080 |
| **Biosynthesis of steroids** | Normal vs. tumor | Tumor | -4.79 | 1.04E-04 | 100 |
| **MAPK signaling pathway** | Normal vs. tumor | Normal | 4.58 | 2.20E-04 | 4010 |
| **Terpenoid biosynthesis** | Normal vs. tumor | Tumor | -4.45 | 2.71E-04 | 900 |
| **Cytokine-cytokine receptor interaction** | Normal vs. tumor | Normal | 4.43 | 2.71E-04 | 4060 |
| **Ribosome** | Normal vs. tumor | Tumor | -4.41 | 2.71E-04 | 3010 |
| **Cholera - Infection** | Normal vs. tumor | Tumor | -4.16 | 7.39E-04 | 5110 |
| **Fatty acid elongation in mitochondria** | Normal vs. tumor | Tumor | -3.91 | 1.87E-03 | 62 |
| **Tyrosine metabolism** | Normal vs. tumor | Normal | 3.81 | 2.62E-03 | 350 |
| **Calcium signaling pathway** | Normal vs. tumor | Normal | 3.55 | 6.51E-03 | 4020 |
| **Complement and coagulation cascades** | Normal vs. tumor | Normal | 3.46 | 8.37E-03 | 4610 |
| **GnRH signaling pathway** | Normal vs. tumor | Normal | 3.39 | 9.82E-03 | 4912 |
| **Hedgehog signaling pathway** | Normal vs. tumor | Normal | 3.05 | 3.06E-02 | 4340 |
| **Taste transduction** | Normal vs. tumor | Normal | 3.01 | 3.25E-02 | 4742 |
| **ECM-receptor interaction** | Normal vs. tumor | Tumor | -2.98 | 3.39E-02 | 4512 |
| **Jak-STAT signaling pathway** | Normal vs. tumor | Normal | 2.96 | 3.40E-02 | 4630 |
| **Long-term depression** | Normal vs. tumor | Normal | 2.88 | 4.12E-02 | 4730 |
| **Proteasome** | Normal vs. tumor | Tumor | -2.84 | 4.34E-02 | 3050 |
|  |  |  |  |  |  |
| **Ribosome** | B vs. A | B | 21.89 | 6.02E-104 | 3010 |
| **Oxidative phosphorylation** | B vs. A | B | 10.40 | 2.19E-23 | 190 |
| **Prion disease** | B vs. A | B | 7.73 | 6.41E-13 | 5060 |
| **Focal adhesion** | B vs. A | B | 7.67 | 7.96E-13 | 4510 |
| **Androgen and estrogen metabolism** | B vs. A | A | -7.23 | 1.74E-11 | 150 |
| **Neuroactive ligand-receptor interaction** | B vs. A | A | -6.18 | 1.91E-08 | 4080 |
| **ECM-receptor interaction** | B vs. A | B | 6.16 | 1.91E-08 | 4512 |
| **Pathogenic Escherichia coli infection - EHEC** | B vs. A | B | 5.98 | 4.61E-08 | 5130 |
| **Pathogenic Escherichia coli infection - EPEC** | B vs. A | B | 5.98 | 4.61E-08 | 5131 |
| **Maturity onset diabetes of the young** | B vs. A | A | -5.63 | 3.33E-07 | 4950 |
| **Metabolism of xenobiotics by cytochrome P450** | B vs. A | A | -4.99 | 9.92E-06 | 980 |
| **Neurodegenerative Disorders** | B vs. A | B | 4.65 | 5.11E-05 | 1510 |
| **Tyrosine metabolism** | B vs. A | A | -4.54 | 7.86E-05 | 350 |
| **Type II diabetes mellitus** | B vs. A | A | -4.53 | 7.94E-05 | 4930 |
| **Pentose and glucuronate interconversions** | B vs. A | A | -4.41 | 1.29E-04 | 40 |
| **T cell receptor signaling pathway** | B vs. A | A | -4.36 | 1.51E-04 | 4660 |
| **Adherens junction** | B vs. A | B | 4.24 | 2.43E-04 | 4520 |
| **Porphyrin and chlorophyll metabolism** | B vs. A | A | -4.20 | 2.76E-04 | 860 |
| **Starch and sucrose metabolism** | B vs. A | A | -4.11 | 3.73E-04 | 500 |
| **Jak-STAT signaling pathway** | B vs. A | A | -4.11 | 3.73E-04 | 4630 |
| **Proteasome** | B vs. A | B | 4.04 | 4.74E-04 | 3050 |
| **Adipocytokine signaling pathway** | B vs. A | A | -4.00 | 5.27E-04 | 4920 |
| **Fc epsilon RI signaling pathway** | B vs. A | A | -3.89 | 8.19E-04 | 4664 |
| **TGF-beta signaling pathway** | B vs. A | B | 3.87 | 8.32E-04 | 4350 |
| **Toll-like receptor signaling pathway** | B vs. A | A | -3.85 | 8.64E-04 | 4620 |
| **Propanoate metabolism** | B vs. A | B | 3.83 | 9.28E-04 | 640 |
| **Cyanoamino acid metabolism** | B vs. A | A | -3.81 | 9.58E-04 | 460 |
| **C21-Steroid hormone metabolism** | B vs. A | A | -3.79 | 1.01E-03 | 140 |
| **Riboflavin metabolism** | B vs. A | A | -3.75 | 1.12E-03 | 740 |
| **Acute myeloid leukemia** | B vs. A | A | -3.68 | 1.41E-03 | 5221 |
| **Dentatorubropallidoluysian atrophy (DRPLA)** | B vs. A | B | 3.67 | 1.41E-03 | 5050 |
| **Phenylalanine, tyrosine and tryptophan biosynthesis** | B vs. A | A | -3.67 | 1.41E-03 | 400 |
| **PPAR signaling pathway** | B vs. A | A | -3.63 | 1.59E-03 | 3320 |
| **Protein export** | B vs. A | B | 3.47 | 2.88E-03 | 3060 |
| **gamma-Hexachlorocyclohexane degradation** | B vs. A | A | -3.42 | 3.32E-03 | 361 |
| **Arachidonic acid metabolism** | B vs. A | A | -3.41 | 3.37E-03 | 590 |
| **Glutamate metabolism** | B vs. A | A | -3.39 | 3.45E-03 | 251 |
| **Atrazine degradation** | B vs. A | A | -3.38 | 3.55E-03 | 791 |
| **Hedgehog signaling pathway** | B vs. A | A | -3.28 | 4.90E-03 | 4340 |
| **ErbB signaling pathway** | B vs. A | A | -3.24 | 5.45E-03 | 4012 |
| **Glutathione metabolism** | B vs. A | A | -3.16 | 7.20E-03 | 480 |
| **ABC transporters - General** | B vs. A | A | -3.05 | 1.01E-02 | 2010 |
| **Natural killer cell mediated cytotoxicity** | B vs. A | A | -3.00 | 1.16E-02 | 4650 |
| **Non-small cell lung cancer** | B vs. A | A | -2.96 | 1.30E-02 | 5223 |
| **Chondroitin sulfate biosynthesis** | B vs. A | B | 2.83 | 1.93E-02 | 532 |
| **Valine, leucine and isoleucine degradation** | B vs. A | B | 2.81 | 2.02E-02 | 280 |
| **2,4-Dichlorobenzoate degradation** | B vs. A | A | -2.77 | 2.22E-02 | 623 |
| **Cell Communication** | B vs. A | B | 2.75 | 2.26E-02 | 1430 |
| **Insulin signaling pathway** | B vs. A | A | -2.73 | 2.37E-02 | 4910 |
| **Cholera - Infection** | B vs. A | B | 2.72 | 2.39E-02 | 5110 |
| **Glycan structures - biosynthesis 2** | B vs. A | A | -2.71 | 2.41E-02 | 1031 |
| **1- and 2-Methylnaphthalene degradation** | B vs. A | A | -2.66 | 2.74E-02 | 624 |
| **Reductive carboxylate cycle (CO2 fixation)** | B vs. A | B | 2.66 | 2.74E-02 | 720 |
| **VEGF signaling pathway** | B vs. A | A | -2.62 | 3.03E-02 | 4370 |
| **Bladder cancer** | B vs. A | A | -2.61 | 3.07E-02 | 5219 |
| **p53 signaling pathway** | B vs. A | A | -2.56 | 3.45E-02 | 4115 |
| **Dorso-ventral axis formation** | B vs. A | A | -2.54 | 3.56E-02 | 4320 |
| **Taste transduction** | B vs. A | A | -2.48 | 4.14E-02 | 4742 |
| **Regulation of autophagy** | B vs. A | A | -2.48 | 4.14E-02 | 4140 |
| **Folate biosynthesis** | B vs. A | A | -2.45 | 4.31E-02 | 790 |
| **Glycolysis / Gluconeogenesis** | B vs. A | B | 2.45 | 4.31E-02 | 10 |
| **Ether lipid metabolism** | B vs. A | A | -2.45 | 4.32E-02 | 565 |
| **Cytokine-cytokine receptor interaction** | B vs. A | A | -2.42 | 4.59E-02 | 4060 |
| **Pantothenate and CoA biosynthesis** | B vs. A | A | -2.38 | 4.93E-02 | 770 |
| **Nitrogen metabolism** | B vs. A | A | -2.38 | 4.93E-02 | 910 |
| **Glycerophospholipid metabolism** | B vs. A | A | -2.37 | 4.93E-02 | 564 |
|  |  |  |  |  |  |
| **Oxidative phosphorylation** | Normal vs. A | A | -7.93 | 4.21E-13 | 190 |
| **Ribosome** | Normal vs. A | Normal | 7.38 | 1.43E-11 | 3010 |
| **Prion disease** | Normal vs. A | Normal | 4.44 | 5.55E-04 | 5060 |
| **Androgen and estrogen metabolism** | Normal vs. A | A | -3.87 | 5.05E-03 | 150 |
| **Biosynthesis of steroids** | Normal vs. A | A | -3.68 | 8.60E-03 | 100 |
| **Pentose and glucuronate interconversions** | Normal vs. A | A | -3.62 | 9.25E-03 | 40 |
| **Porphyrin and chlorophyll metabolism** | Normal vs. A | A | -3.47 | 1.38E-02 | 860 |
| **MAPK signaling pathway** | Normal vs. A | Normal | 3.28 | 2.37E-02 | 4010 |
| **Sulfur metabolism** | Normal vs. A | A | -3.20 | 2.72E-02 | 920 |
| **Terpenoid biosynthesis** | Normal vs. A | A | -3.18 | 2.72E-02 | 900 |
| **Pantothenate and CoA biosynthesis** | Normal vs. A | A | -3.07 | 3.62E-02 | 770 |
| **Arginine and proline metabolism** | Normal vs. A | A | -3.00 | 4.11E-02 | 330 |
| **Neurodegenerative Disorders** | Normal vs. A | Normal | 2.97 | 4.11E-02 | 1510 |
| **Focal adhesion** | Normal vs. A | Normal | 2.96 | 4.11E-02 | 4510 |
| **2,4-Dichlorobenzoate degradation** | Normal vs. A | A | -2.91 | 4.52E-02 | 623 |
|  |  |  |  |  |  |
| **Ribosome** | Normal vs. B | B | -17.88 | 3.36E-69 | 3010 |
| **Oxidative phosphorylation** | Normal vs. B | B | -17.72 | 2.59E-68 | 190 |
| **Neuroactive ligand-receptor interaction** | Normal vs. B | Normal | 7.98 | 8.73E-14 | 4080 |
| **ECM-receptor interaction** | Normal vs. B | B | -6.37 | 8.71E-09 | 4512 |
| **Tyrosine metabolism** | Normal vs. B | Normal | 5.94 | 1.08E-07 | 350 |
| **Maturity onset diabetes of the young** | Normal vs. B | Normal | 5.64 | 5.06E-07 | 4950 |
| **Pathogenic Escherichia coli infection - EHEC** | Normal vs. B | B | -5.60 | 5.06E-07 | 5130 |
| **Pathogenic Escherichia coli infection - EPEC** | Normal vs. B | B | -5.60 | 5.06E-07 | 5131 |
| **Cytokine-cytokine receptor interaction** | Normal vs. B | Normal | 5.25 | 3.09E-06 | 4060 |
| **Focal adhesion** | Normal vs. B | B | -5.22 | 3.30E-06 | 4510 |
| **Cholera - Infection** | Normal vs. B | B | -5.00 | 9.77E-06 | 5110 |
| **Jak-STAT signaling pathway** | Normal vs. B | Normal | 4.98 | 9.83E-06 | 4630 |
| **Metabolism of xenobiotics by cytochrome P450** | Normal vs. B | Normal | 4.82 | 2.00E-05 | 980 |
| **Proteasome** | Normal vs. B | B | -4.81 | 2.00E-05 | 3050 |
| **Biosynthesis of steroids** | Normal vs. B | B | -4.71 | 3.07E-05 | 100 |
| **Terpenoid biosynthesis** | Normal vs. B | B | -4.67 | 3.49E-05 | 900 |
| **Type II diabetes mellitus** | Normal vs. B | Normal | 4.59 | 4.85E-05 | 4930 |
| **Fatty acid elongation in mitochondria** | Normal vs. B | B | -4.53 | 6.03E-05 | 62 |
| **Hedgehog signaling pathway** | Normal vs. B | Normal | 4.47 | 7.38E-05 | 4340 |
| **Prion disease** | Normal vs. B | B | -4.47 | 7.38E-05 | 5060 |
| **MAPK signaling pathway** | Normal vs. B | Normal | 4.42 | 8.63E-05 | 4010 |
| **Androgen and estrogen metabolism** | Normal vs. B | Normal | 4.36 | 1.11E-04 | 150 |
| **PPAR signaling pathway** | Normal vs. B | Normal | 4.26 | 1.63E-04 | 3320 |
| **Toll-like receptor signaling pathway** | Normal vs. B | Normal | 4.12 | 2.96E-04 | 4620 |
| **ABC transporters - General** | Normal vs. B | Normal | 3.92 | 6.46E-04 | 2010 |
| **Taste transduction** | Normal vs. B | Normal | 3.90 | 6.74E-04 | 4742 |
| **Acute myeloid leukemia** | Normal vs. B | Normal | 3.82 | 9.29E-04 | 5221 |
| **GnRH signaling pathway** | Normal vs. B | Normal | 3.79 | 9.84E-04 | 4912 |
| **Adipocytokine signaling pathway** | Normal vs. B | Normal | 3.71 | 1.34E-03 | 4920 |
| **Hematopoietic cell lineage** | Normal vs. B | Normal | 3.61 | 1.88E-03 | 4640 |
| **Arachidonic acid metabolism** | Normal vs. B | Normal | 3.51 | 2.65E-03 | 590 |
| **Complement and coagulation cascades** | Normal vs. B | Normal | 3.45 | 3.25E-03 | 4610 |
| **Chondroitin sulfate biosynthesis** | Normal vs. B | B | -3.43 | 3.30E-03 | 532 |
| **ErbB signaling pathway** | Normal vs. B | Normal | 3.43 | 3.30E-03 | 4012 |
| **Calcium signaling pathway** | Normal vs. B | Normal | 3.39 | 3.73E-03 | 4020 |
| **Dorso-ventral axis formation** | Normal vs. B | Normal | 3.30 | 5.00E-03 | 4320 |
| **Phenylalanine, tyrosine and tryptophan biosynthesis** | Normal vs. B | Normal | 3.28 | 5.21E-03 | 400 |
| **Cyanoamino acid metabolism** | Normal vs. B | Normal | 3.26 | 5.40E-03 | 460 |
| **Taurine and hypotaurine metabolism** | Normal vs. B | Normal | 3.24 | 5.74E-03 | 430 |
| **gamma-Hexachlorocyclohexane degradation** | Normal vs. B | Normal | 3.21 | 6.24E-03 | 361 |
| **Ether lipid metabolism** | Normal vs. B | Normal | 3.15 | 7.43E-03 | 565 |
| **Fc epsilon RI signaling pathway** | Normal vs. B | Normal | 3.11 | 8.31E-03 | 4664 |
| **Long-term depression** | Normal vs. B | Normal | 3.01 | 1.13E-02 | 4730 |
| **Natural killer cell mediated cytotoxicity** | Normal vs. B | Normal | 2.87 | 1.70E-02 | 4650 |
| **T cell receptor signaling pathway** | Normal vs. B | Normal | 2.83 | 1.86E-02 | 4660 |
| **Glycine, serine and threonine metabolism** | Normal vs. B | Normal | 2.83 | 1.86E-02 | 260 |
| **Inositol phosphate metabolism** | Normal vs. B | Normal | 2.80 | 2.02E-02 | 562 |
| **Valine, leucine and isoleucine degradation** | Normal vs. B | B | -2.74 | 2.34E-02 | 280 |
| **Endometrial cancer** | Normal vs. B | Normal | 2.73 | 2.42E-02 | 5213 |
| **Alzheimer's disease** | Normal vs. B | B | -2.72 | 2.44E-02 | 5010 |
| **Reductive carboxylate cycle (CO2 fixation)** | Normal vs. B | B | -2.65 | 2.88E-02 | 720 |
| **Nitrogen metabolism** | Normal vs. B | Normal | 2.64 | 2.91E-02 | 910 |
| **Olfactory transduction** | Normal vs. B | Normal | 2.63 | 2.97E-02 | 4740 |
| **Protein export** | Normal vs. B | B | -2.61 | 3.13E-02 | 3060 |
| **Glycerophospholipid metabolism** | Normal vs. B | Normal | 2.60 | 3.18E-02 | 564 |
| **VEGF signaling pathway** | Normal vs. B | Normal | 2.56 | 3.41E-02 | 4370 |
| **Cell Communication** | Normal vs. B | B | -2.54 | 3.56E-02 | 1430 |
| **Dentatorubropallidoluysian atrophy (DRPLA)** | Normal vs. B | B | -2.47 | 4.37E-02 | 5050 |
| **1- and 2-Methylnaphthalene degradation** | Normal vs. B | Normal | 2.44 | 4.63E-02 | 624 |
| **Folate biosynthesis** | Normal vs. B | Normal | 2.41 | 4.95E-02 | 790 |

**Supplementary Table S3: List of genes differentially expressed between samples from recurrent and non-recurrent patients.** Probes with positive fold changes have higher expression in patients with recurrent disease.

| Probe name | Fold change | P-value | Gene symbol | Gene name | | |
| --- | --- | --- | --- | --- | --- | --- |
| A_23_P122662 | 4.49 | 1.14E-04 | GFOD1 | glucose-fructose oxidoreductase domain containing 1 | | |
| A_23_P217899 | 2.16 | 1.68E-04 | CCNL2 | cyclin L2 | | |
| A_23_P25155 | -2.49 | 2.71E-04 | GPR84 | G protein-coupled receptor 84 | | |
| A_23_P151027 | 1.67 | 3.36E-04 | KIAA0528 | KIAA0528 | | |
| A_23_P350754 | -2.47 | 3.41E-04 | OR7E13P | olfactory receptor, family 7, subfamily E, member 13 pseudogene | | |
| A_23_P152272 | 1.57 | 4.19E-04 | RNPS1 | R binding protein S1, serine-rich domain | | |
| A_32_P215856 | -2.76 | 5.65E-04 | A_32_P215856 |  | | |
| A_24_P161367 | -3.62 | 5.66E-04 | AK027158 |  | | |
| A_32_P231250 | -2.96 | 6.19E-04 | AI085826 |  | | |
| A_23_P202374 | -4.41 | 6.82E-04 | COX15 | COX15 homolog, cytochrome c oxidase assembly protein (yeast) | | |
| A_23_P124619 | -2.95 | 7.85E-04 | S100A14 | S100 calcium binding protein A14 | | |
| A_23_P250196 | 1.91 | 8.16E-04 | MTDH | metadherin | | |
| A_23_P131337 | 1.71 | 1.06E-03 | SMARCAL1 | SWI/SNF related, matrix associated, actin dependent regulator of chromatin, subfamily a-like 1 | | |
| A_23_P92499 | -4.13 | 1.27E-03 | TLR2 | toll-like receptor 2 | | |
| A_32_P32391 | -2.39 | 1.34E-03 | OR7E156P | olfactory receptor, family 7, subfamily E, member 156 pseudogene | | |
| A_24_P944144 | 3.03 | 1.36E-03 | DATF1 | death inducer-obliterator 1 | | |
| A_23_P117225 | 1.75 | 1.42E-03 | ERCC5 | excision repair cross-complementing rodent repair deficiency, complementation group 5 (xeroderma pigmentosum, complementation group G (Cockayne syndrome)) | | |
| A_23_P155477 | 2.28 | 1.47E-03 | C3orf18 | chromosome 3 open reading frame 18 | | |
| A_24_P132039 | 1.81 | 1.84E-03 | RNF14 | ring finger protein 14 | | |
| A_23_P86917 | -2.17 | 1.91E-03 | FADD | Fas (TNFRSF6)-associated via death domain | | |
| A_24_P84711 | 1.46 | 1.96E-03 | A_24_P84711 |  | | |
| A_23_P48416 | 1.89 | 1.97E-03 | DIS3 | DIS3 mitotic control homolog (S. cerevisiae) | | |
| A_23_P321920 | 1.40 | 2.00E-03 | CCL3L3 | chemokine (C-C motif) ligand 3-like 3 | | |
| A_24_P373844 | -2.52 | 2.24E-03 | THC2276504 |  | | |
| A_24_P400970 | 1.81 | 2.29E-03 | A_24_P400970 |  | | |
| A_23_P164451 | 2.75 | 2.56E-03 | TBX2 | T-box 2 | | |
| A_32_P59355 | -1.80 | 2.67E-03 | THC2343897 |  | | |
| A_32_P185507 | 1.84 | 2.71E-03 | PMS2L1 | postmeiotic segregation increased 2-like 1 | | |
| A_23_P351232 | 1.38 | 2.73E-03 | ZNF775 | zinc finger protein 775 | | |
| A_24_P669220 | 1.73 | 2.73E-03 | THC2314754 |  | | |
| A_24_P455060 | 1.95 | 2.98E-03 | CR616058 | similar to FUS interacting protein (serine-arginine rich) 1 | | |
| A_23_P253602 | 1.82 | 3.02E-03 | BMX | BMX non-receptor tyrosine kise | | |
| A_23_P36266 | 1.68 | 3.05E-03 | PDHX | pyruvate dehydrogese complex, component X | | |
| A_23_P344531 | 1.97 | 3.14E-03 | SYNPO | syptopodin | | |
| A_32_P144920 | 1.53 | 3.15E-03 | NECAP1 | NECAP endocytosis associated 1 | | |
| A_23_P140654 | -5.14 | 3.16E-03 | THC2375798 |  | | |
| A_32_P167904 | -2.07 | 3.29E-03 | CR624679 |  | | |
| A_24_P639441 | 1.52 | 3.36E-03 | CD59 | CD59 molecule, complement regulatory protein | | |
| A_24_P297078 | 1.72 | 3.50E-03 | C20orf3 | chromosome 20 open reading frame 3 | | |
| A_23_P127079 | 1.96 | 3.52E-03 | PPRC1 | peroxisome proliferator-activated receptor gamma, coactivator-related 1 | | |
| A_23_P314086 | 2.19 | 3.56E-03 | RNF126 | ring finger protein 126 | | |
| A_23_P122387 | 1.64 | 3.60E-03 | MRPS18A | mitochondrial ribosomal protein S18A | | |
| A_23_P207999 | 1.54 | 3.66E-03 | PMAIP1 | phorbol-12-myristate-13-acetate-induced protein 1 | | |
| A_32_P224638 | 1.59 | 3.78E-03 | THC2407541 |  | | |
| A_32_P78904 | 2.10 | 3.91E-03 | THC2312785 |  | | |
| A_24_P255836 | -2.02 | 4.01E-03 | A_24_P255836 |  | | |
| A_23_P250196 | 1.71 | 4.04E-03 | MTDH | metadherin | | |
| A_32_P105825 | -1.90 | 4.28E-03 | MPPED2 | metallophosphoesterase domain containing 2 | | |
| A_23_P433820 | 1.43 | 4.34E-03 | THAP8 | THAP domain containing 8 | | |
| A_24_P28722 | -4.63 | 4.68E-03 | RSAD2 | radical S-adenosyl methionine domain containing 2 | | |
| A_23_P205778 | 2.06 | 5.01E-03 | GNB5 | guanine nucleotide binding protein (G protein), beta 5 | | |
| A_23_P204364 | 1.72 | 5.11E-03 | NOL1 | | nucleolar protein 1, 120kDa | |
| A_23_P336218 | 1.67 | 5.11E-03 | MGC27345 | | hypothetical protein MGC27345 | |
| A_24_P130026 | -3.66 | 5.18E-03 | BAG5 | | BCL2-associated athanogene 5 | |
| A_23_P116286 | -2.51 | 5.23E-03 | AMPD3 | | adenosine monophosphate deamise (isoform E) | |
| A_24_P290013 | 1.61 | 5.28E-03 | ENST00000338711 | | | |
| A_23_P130089 | 1.87 | 5.36E-03 | IFT20 | | | intraflagellar transport 20 homolog (Chlamydomos) |
| A_24_P394569 | 2.16 | 5.39E-03 | AK056855 | | |  |
| A_32_P86245 | -1.77 | 5.52E-03 | EFHC1 | | | EF-hand domain (C-termil) containing 1 |
| A_24_P376339 | -2.93 | 5.60E-03 | CCNL2 | | | cyclin L2 |
| A_23_P18505 | -3.31 | 5.67E-03 | GAB1 | | | GRB2-associated binding protein 1 |
| A_23_P25433 | -4.80 | 5.68E-03 | C12orf4 | | | chromosome 12 open reading frame 4 |
| A_23_P845 | -1.86 | 5.72E-03 | TOMM40L | | | translocase of outer mitochondrial membrane 40 homolog (yeast)-like |
| A_24_P299685 | 1.46 | 5.81E-03 | PDPN | | | podoplanin |
| A_23_P37205 | -1.92 | 5.84E-03 | NDRG2 | | | NDRG family member 2 |
| A_32_P113380 | 2.54 | 6.21E-03 | THC2411712 | | |  |
| A_23_P111621 | -2.08 | 6.23E-03 | GTF2IRD1 | | | GTF2I repeat domain containing 1 |
| A_23_P207999 | 1.56 | 6.34E-03 | PMAIP1 | | | phorbol-12-myristate-13-acetate-induced protein 1 |
| A_24_P303127 | -5.72 | 6.36E-03 | C5orf29 | | | chromosome 5 open reading frame 29 |
| A_23_P204158 | -2.19 | 6.43E-03 | TMEM118 | | | transmembrane protein 118 |
| A_23_P63668 | -3.91 | 6.48E-03 | IFIT5 | | | interferon-induced protein with tetratricopeptide repeats 5 |
| A_23_P426196 | 1.60 | 6.49E-03 | MAST3 | | | microtubule associated serine/threonine kise 3 |
| A_23_P123424 | 1.70 | 6.56E-03 | CHRNB3 | | | cholinergic receptor, nicotinic, beta 3 |
| A_23_P250196 | 1.68 | 6.61E-03 | MTDH | | | metadherin |
| A_23_P40078 | -2.96 | 6.62E-03 | XPO1 | | | exportin 1 (CRM1 homolog, yeast) |
| A_24_P212677 | 1.67 | 6.63E-03 | RNF170 | | | ring finger protein 170 |
| A_24_P184937 | 1.79 | 6.66E-03 | C16orf44 | | | chromosome 16 open reading frame 44 |
| A_32_P125542 | -1.88 | 6.67E-03 | THC2384778 | | |  |
| A_23_P121602 | -3.10 | 6.77E-03 | SAP30 | | | Sin3A-associated protein, 30kDa |
| A_24_P343830 | 1.69 | 6.79E-03 | FLJ22596 | | | hypothetical protein FLJ22596 |
| A_23_P135123 | 1.52 | 6.91E-03 | FRMD3 | | | FERM domain containing 3 |
| A_32_P107463 | -1.53 | 6.98E-03 | EFCAB2 | | | EF-hand calcium binding domain 2 |
| A_32_P120977 | 1.65 | 7.02E-03 | PX19 | | | PRELI domain containing 1 |
| A_23_P200396 | -1.83 | 7.10E-03 | C1orf91 | | | chromosome 1 open reading frame 91 |
| A_23_P55601 | -2.73 | 7.11E-03 | ZNF236 | | | zinc finger protein 236 |
| A_23_P8906 | -4.81 | 7.12E-03 | LRP12 | | | low density lipoprotein-related protein 12 |
| A_23_P12044 | -2.57 | 7.12E-03 | TMEM51 | | | transmembrane protein 51 |
| A_23_P405942 | 1.53 | 7.12E-03 | LARP5 | | | La ribonucleoprotein domain family, member 5 |
| A_32_P222515 | 1.43 | 7.53E-03 | THC2439829 | | |  |
| A_24_P316305 | 2.47 | 7.60E-03 | AQR | | | aquarius homolog (mouse) |
| A_24_P807031 | 3.23 | 7.75E-03 | LOC92270 | | | hypothetical protein LOC92270 |
| A_32_P14243 | 2.14 | 7.83E-03 | THC2439490 | | |  |
| A_24_P253755 | -1.66 | 7.94E-03 | PIGL | | | phosphatidylinositol glycan anchor biosynthesis, class L |
| A_24_P230009 | 1.63 | 7.98E-03 | A_24_P230009 | | |  |
| A_32_P129894 | 1.51 | 8.11E-03 | EGFL5 | | | multiple EGF-like-domains 9 |
| A_24_P386323 | 1.91 | 8.14E-03 | RABEPK | | | Rab9 effector protein with kelch motifs |
| A_23_P203949 | 1.60 | 8.23E-03 | DDX11 | | | DEAD/H (Asp-Glu-Ala-Asp/His) box polypeptide 11 (CHL1-like helicase homolog, S. cerevisiae) |
| A_32_P157945 | -2.86 | 8.28E-03 | DSP | | | desmoplakin |
| A_24_P141688 | 1.76 | 8.31E-03 | PCBP2 | | | poly(rC) binding protein 2 |
| A_24_P59387 | 2.06 | 8.39E-03 | C8orf60 | | | chromosome 8 open reading frame 60 |
| A_24_P455972 | -1.58 | 8.40E-03 | CR622681 | | | hypothetical protein FLJ43663 |
| A_23_P117286 | 2.43 | 8.61E-03 | CLN5 | | | ceroid-lipofuscinosis, neurol 5 |
| A_23_P66473 | 1.56 | 8.67E-03 | PITPNC1 | | | phosphatidylinositol transfer protein, cytoplasmic 1 |
| A_23_P123234 | 1.62 | 8.72E-03 | A_23_P123234 | | |  |
| A_23_P399255 | 1.79 | 8.74E-03 | RNF182 | | | ring finger protein 182 |
| A_23_P130919 | 1.40 | 8.90E-03 | MOBKL2A | | | MOB1, Mps One Binder kise activator-like 2A (yeast) |
| A_24_P227141 | -3.23 | 9.01E-03 | ELF5 | | | E74-like factor 5 (ets domain transcription factor) |
| A_23_P104641 | 2.19 | 9.02E-03 | C11orf2 | | | chromosome 11 open reading frame2 |
| A_23_P155441 | 1.34 | 9.07E-03 | RFT1 | | | RFT1 homolog (S. cerevisiae) |
| A_23_P167269 | 1.96 | 9.08E-03 | FLJ11184 | | | hypothetical protein FLJ11184 |
| A_23_P61524 | -1.38 | 9.19E-03 | CCDC71 | | | coiled-coil domain containing 71 |
| A_23_P324754 | -1.97 | 9.27E-03 | KIAA1199 | | | KIAA1199 |
| A_32_P218734 | 3.34 | 9.44E-03 | COL5A2 | | | collagen, type V, alpha 2 |
| A_24_P492885 | 1.89 | 9.46E-03 | A_24_P492885 | | |  |
| A_32_P95960 | -2.26 | 9.46E-03 | CLEC2D | | | C-type lectin domain family 2, member D |
| A_23_P123234 | 1.61 | 9.52E-03 | A_23_P123234 | | |  |
| A_24_P142973 | -1.71 | 9.61E-03 | PEX26 | | | peroxisome biogenesis factor 26 |
| A_24_P372223 | -3.33 | 9.61E-03 | MSR1 | | | macrophage scavenger receptor 1 |
| A_32_P221748 | -2.99 | 9.69E-03 | BX350880 | | |  |
| A_23_P20704 | -1.79 | 9.73E-03 | IFNA10 | | | interferon, alpha 10 |
| A_32_P1360 | 1.87 | 9.77E-03 | THC2314369 | | |  |
| A_23_P43412 | 2.15 | 9.78E-03 | HEMGN | | | hemogen |
| A_23_P58137 | -3.36 | 9.82E-03 | ARL9 | | |  |
| A_23_P211428 | 1.46 | 9.86E-03 | SMTN | | | smoothelin |
| A_23_P104295 | 1.31 | 9.88E-03 | SHFM3 | | | F-box and WD repeat domain containing 4 |
| A_23_P123234 | 1.59 | 9.89E-03 | A_23_P123234 | | |  |
| A_32_P178842 | 2.16 | 9.91E-03 | BM906215 | | |  |
| A_23_P374310 | 1.45 | 9.95E-03 | TAC4 | | | tachykinin 4 (hemokinin) |
