# Supplementary material for: Gene-expression profiling of microdissected breast cancer microvasculature identifies distinct tumor vascular subtypes
Source: Breast Cancer Res. 2012 Aug 20;14(4):R120. doi: 10.1186/bcr3246 (PMC3680943; doi:10.1186/bcr3246)

Additional File 2 - Supplementary Figures S1-S6

Supplementary Figure S1. Specific enrichment of the vasculature by laser capture microdissection. A: Representative sections of breast tumor samples with high or low PECAM1 density. Scale bar, 500 µm. B: Representative images of vascular cells stained for PECAM1 before LCM, remaining tissue after LCM, and cells collected by LCM. C: Relative expression of vascular (PECAM1) and epithelial (KRT8) markers in vascular (n=22) and matched epithelial (n=7) samples collected by LCM, as determined by qRTPCR.

Supplementary Figure S2. A: Heatmap depicting differential gene expression following class discovery within the set of all microdissected endothelial samples, performed by hierarchical clustering using Ward's minimum variance method with a correlation distance metric. B: Principal component analysis (PCA) of tumor endothelial samples, based on the 500 genes with highest variance. The first principal component (PC1) accounts for 34.2% of the total variance; the second principal component (PC2) accounts for 15.4%. Colors of points denote sample vascular subtype assignments as in Figure 1A.

Supplementary Figure S3. A: Characteristics of the studies in which other tumor vascular signatures were generated. B: Heatmap representing expression of anti-angiogenic genes in tumor vasculature, showing increased expression in the B subtype. C: qRT-PCR and microarray-derived expression of the pericyte marker PDGFRβ, demonstrating subtype-specific differential expression. qRT-PCR was performed on 4 subtype A and 6 subtype B tumor vasculature samples. The units are relative to the mean of the A subtype; error bars represent standard error. D-G: Heatmaps of tumor vascular signature from St-Croix *et al.* (D), Buckanovich *et al.* (E), Pen *et al.* (F) and Madden *et al.* (G) (5,6,11,12) separate the subtype B tumor vasculature from the remaining samples, but fail to segregate the subtype A tumor vasculature from the normal samples.

Supplementary Figure S4. A: Anti-ACTA2 IHC. Each image is of a representative 1 mm x 1 mm area; inset depicts whole section; scale bar in inset, 1 mm. B, C: Proximity

of ACTA2/LAMB1 and PECAM1 immunostaining. Anti-ACTA2 (B) or anti-LAMB1 (C) and anti-PECAM1 IHC, respectively, was carried out on consecutive sections. Each image is of a representative 0.5 mm x 0.5 mm area. For all panels, individual sample labels are indicated in italic font in the upper left corner of each image.

Supplementary Figure S5. Validation for selected genes identified from analysis of microarray data. qRT-PCR was performed on 4 subtype A and 6 subtype B tumor vasculature samples. Graphs depict regression of fold change in array expression vs. fold change in qRT-PCR-derived concentration for selected genes (ANGPT2, FADD, FAM8A1, GNG10, LAMB1, LYVE1, MET, PDGFRB, SFRP2, SPARC, TLR2 and TFF3). Axes represent fold changes for microarray- and qRT-PCR-derived values, relative to the lowest-expressing sample observed in the array data. The P-value is the significance of the slope term in the regression model, calculated using the lm module in R.

Supplementary Figure S6. Analyses of correlation between cell type marker content and expression of genes differentially expressed between the A and B subtypes. A: Heatmap depicting pericyte marker gene expression across all microdissected endothelial samples; samples are ordered by relative expression of these markers. Each gene in the pericyte signature was annotated with either a positive or negative direction (positive, genes over-expressed in pericytes; negative, genes under-expressed in pericytes). Samples were then linearly ordered from left to right according to the total sum of absolute expression over all genes in the pericyte signature. B: Heatmap depicting expression of the top 200 genes identified as differentially expressed between the A and B subtypes, ranked by P-value (Additional File 4, Dataset S2A), across all endothelial samples. Sample ordering along the horizontal axis is fixed as that determined by relative pericyte marker gene expression in A, above. C: Heatmap depicting expression of endothelial marker genes across all endothelial samples and B subtypes, ranked by P-value. Sample ordering is fixed as in A and B above. D: Heatmap depicting pericyte marker gene expression across all tumor endothelial samples of subtype B. Samples are ordered by pericyte marker gene expression using the method described in A above. E: Heatmap depicting expression of

the top 200 genes identified as differentially expressed between the A and B subtypes (ranked by P-value), across all subtype B tumor endothelial samples. Sample ordering (horizontal axis) is fixed as that determined by relative pericyte marker gene expression in D, above.

Supplementary Figure 1

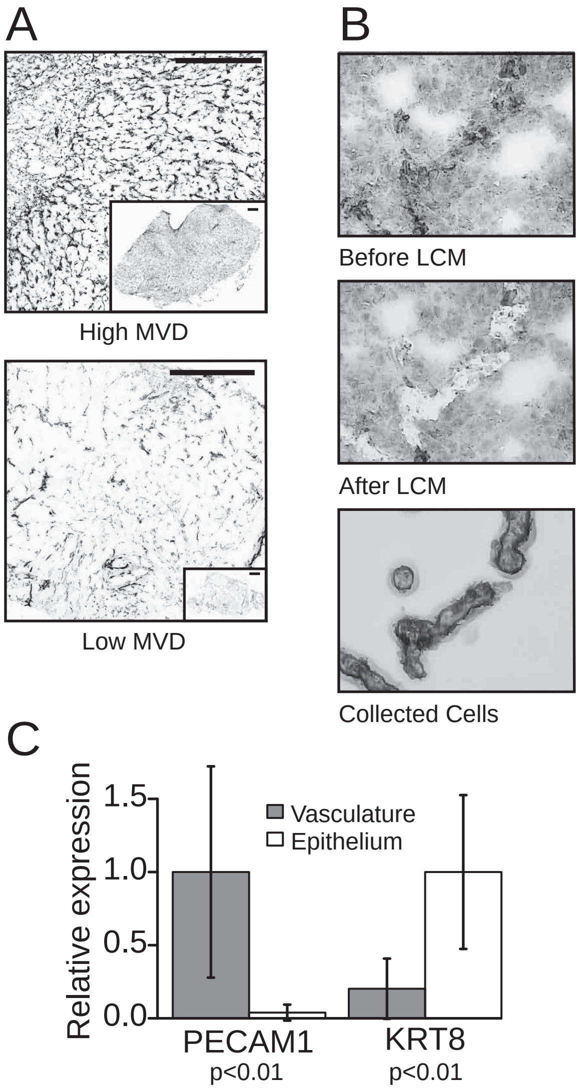

Supplementary Figure 2

A

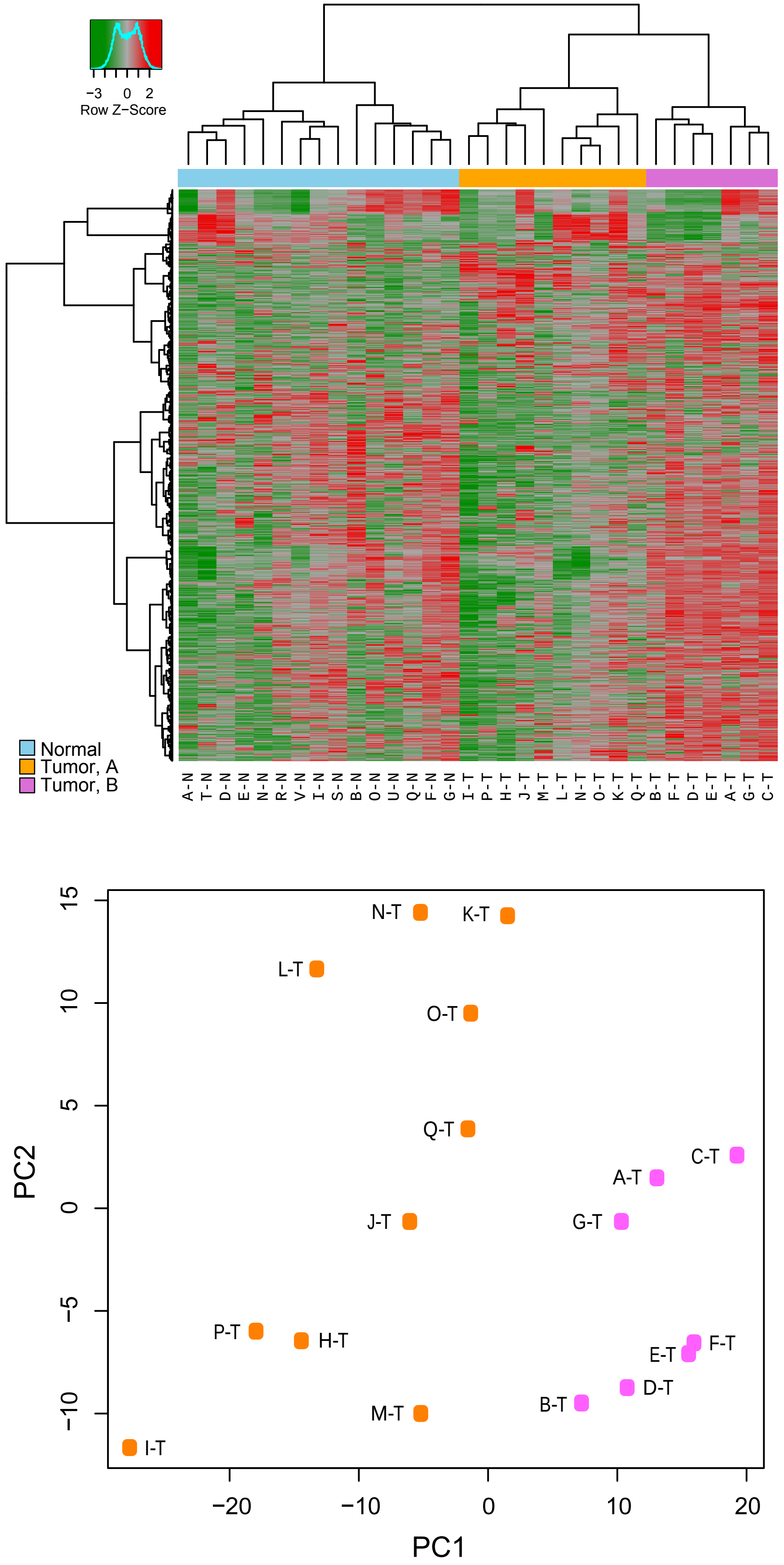

Supplementary Figure 3

A

Study	Origin	Isolation technique	n	Gene overlap	Overlap p
Bhati et al.	Breast	LCM	5	13/51	1e-10
Parker et al.	Breast	Magnetic beads	2	4/26	0.003
St-Croix et al.	Colon	Magnetic beads	1	4/22	0.002
Van Beijnum et al.	Colon	Magnetic beads	5	2/15	0.05
Buckanovich et al.	Ovary	LCM	21	2/12	0.03
Pen et al.	Brain	LCM	5	5/43	0.003
Madden et al.	Brain	Magnetic beads	5	7/143	0.05

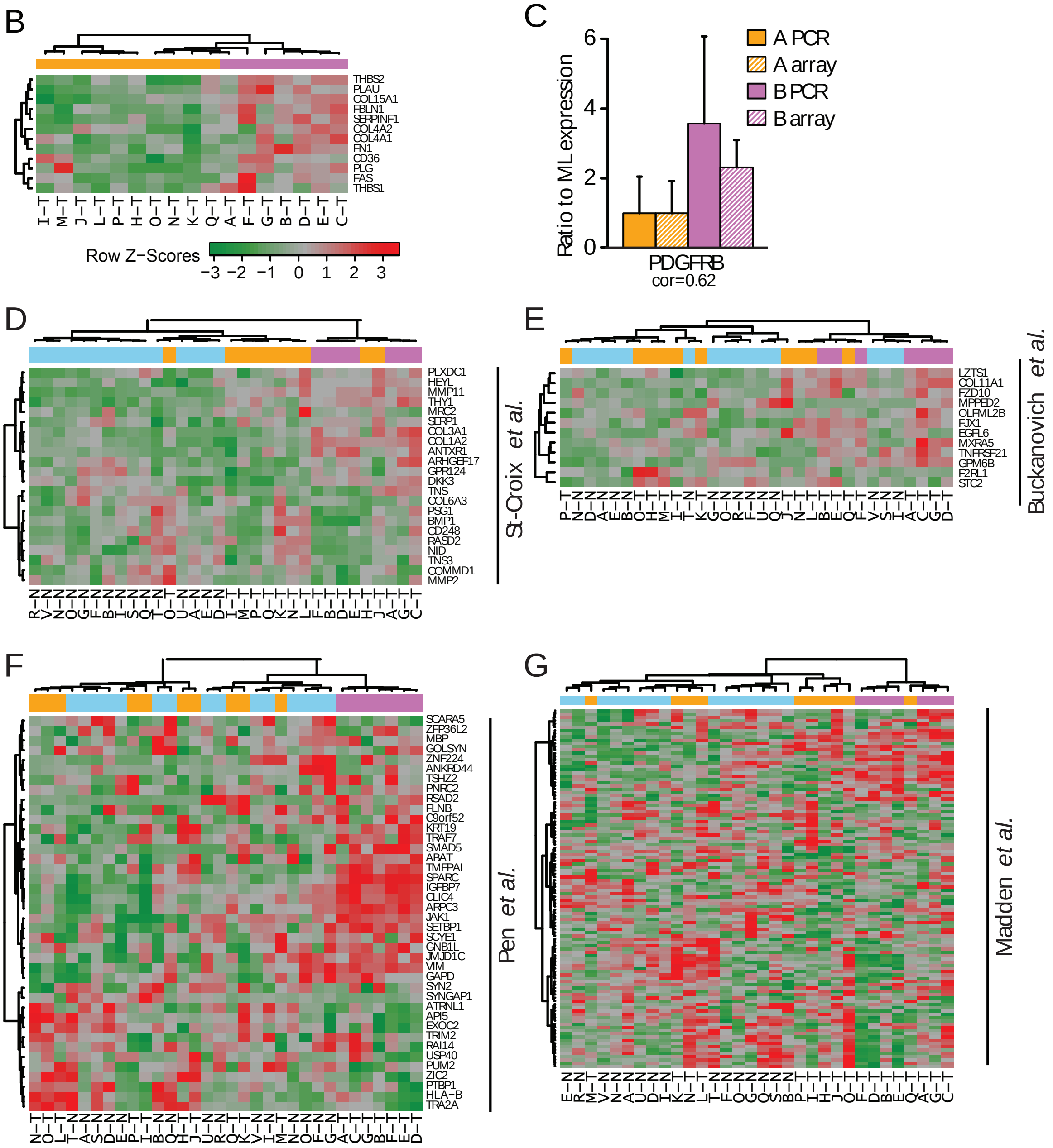

Supplementary Figure 4

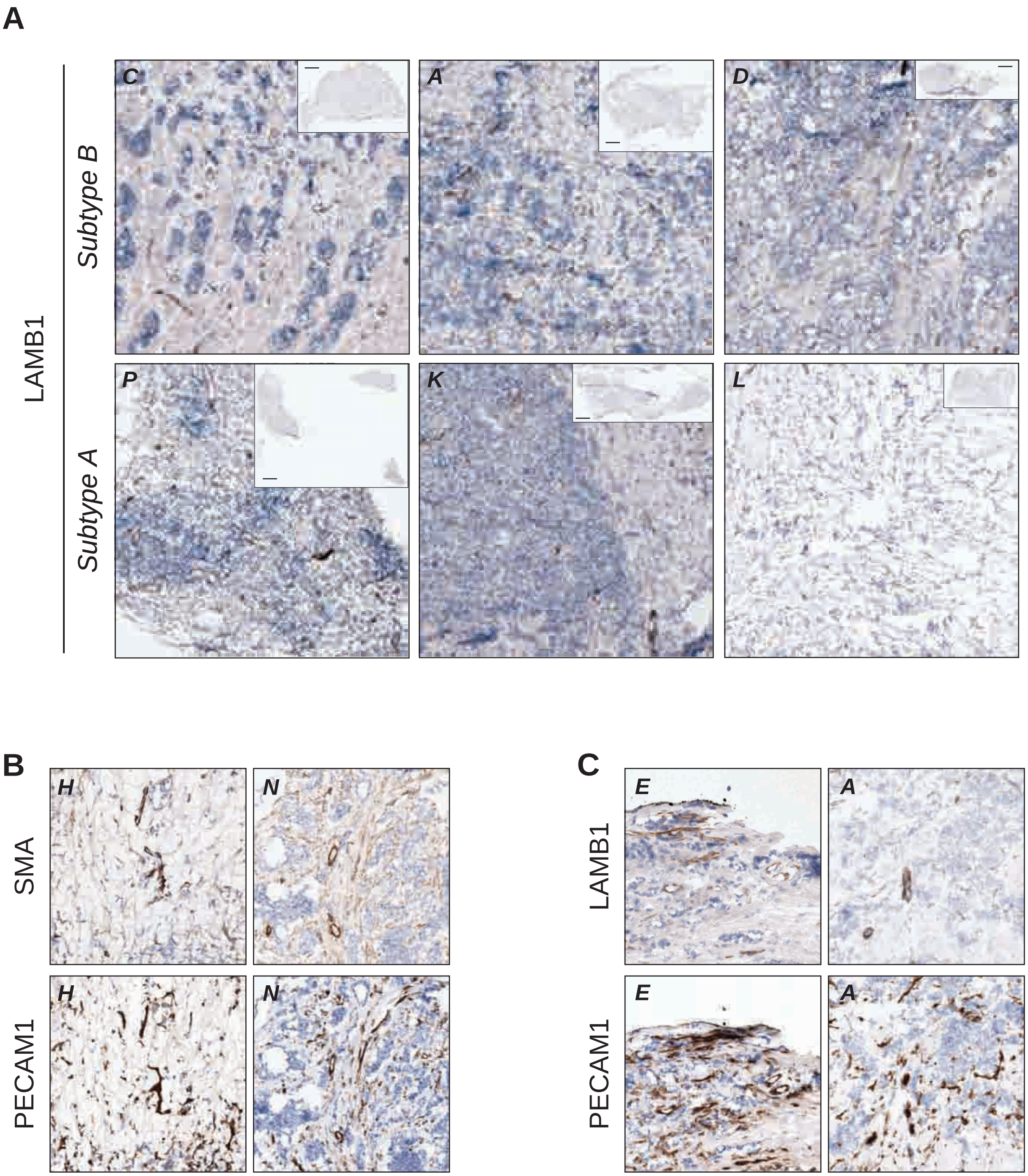

Supplementary Figure 5

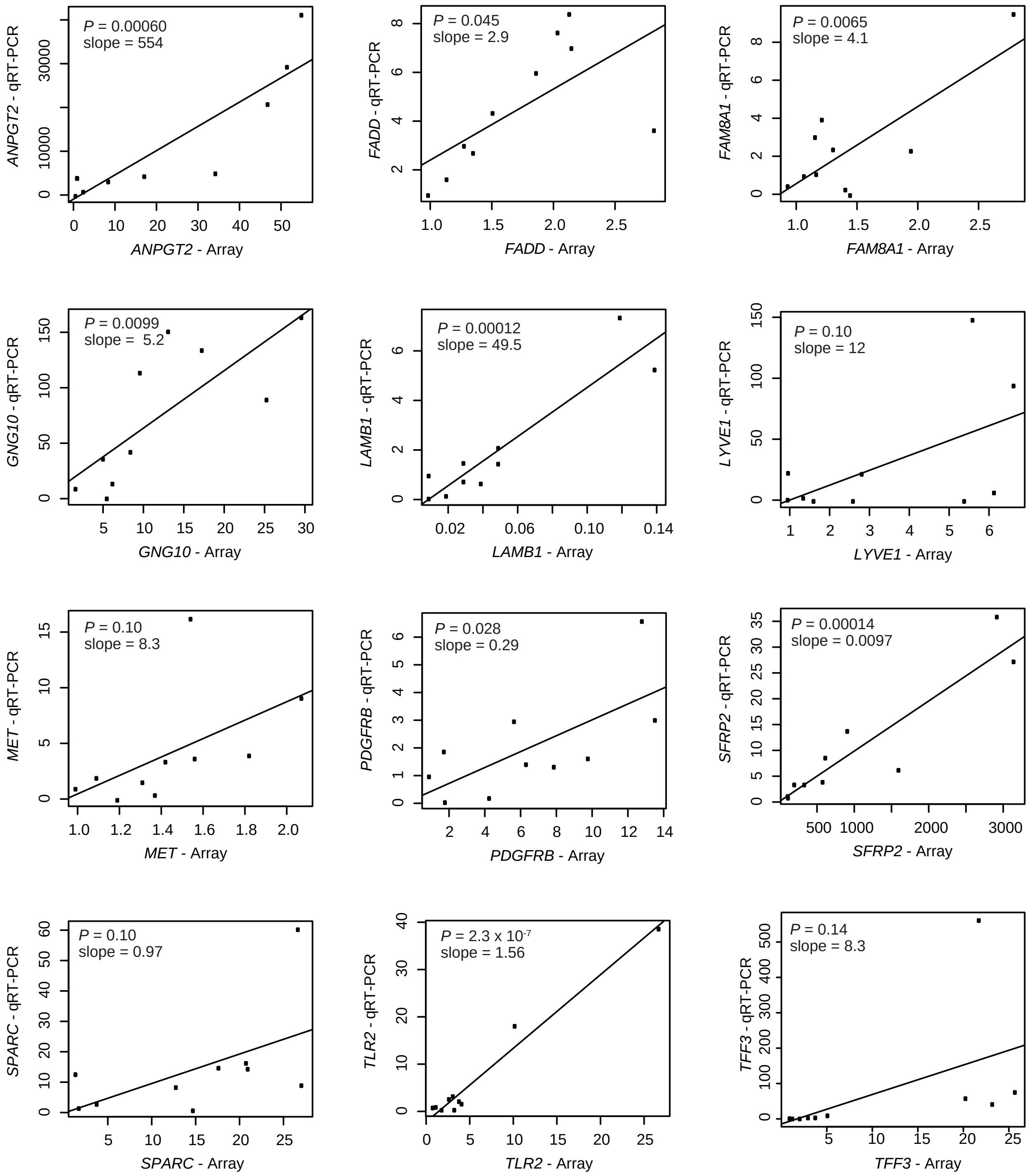

Supplementary Figure 6

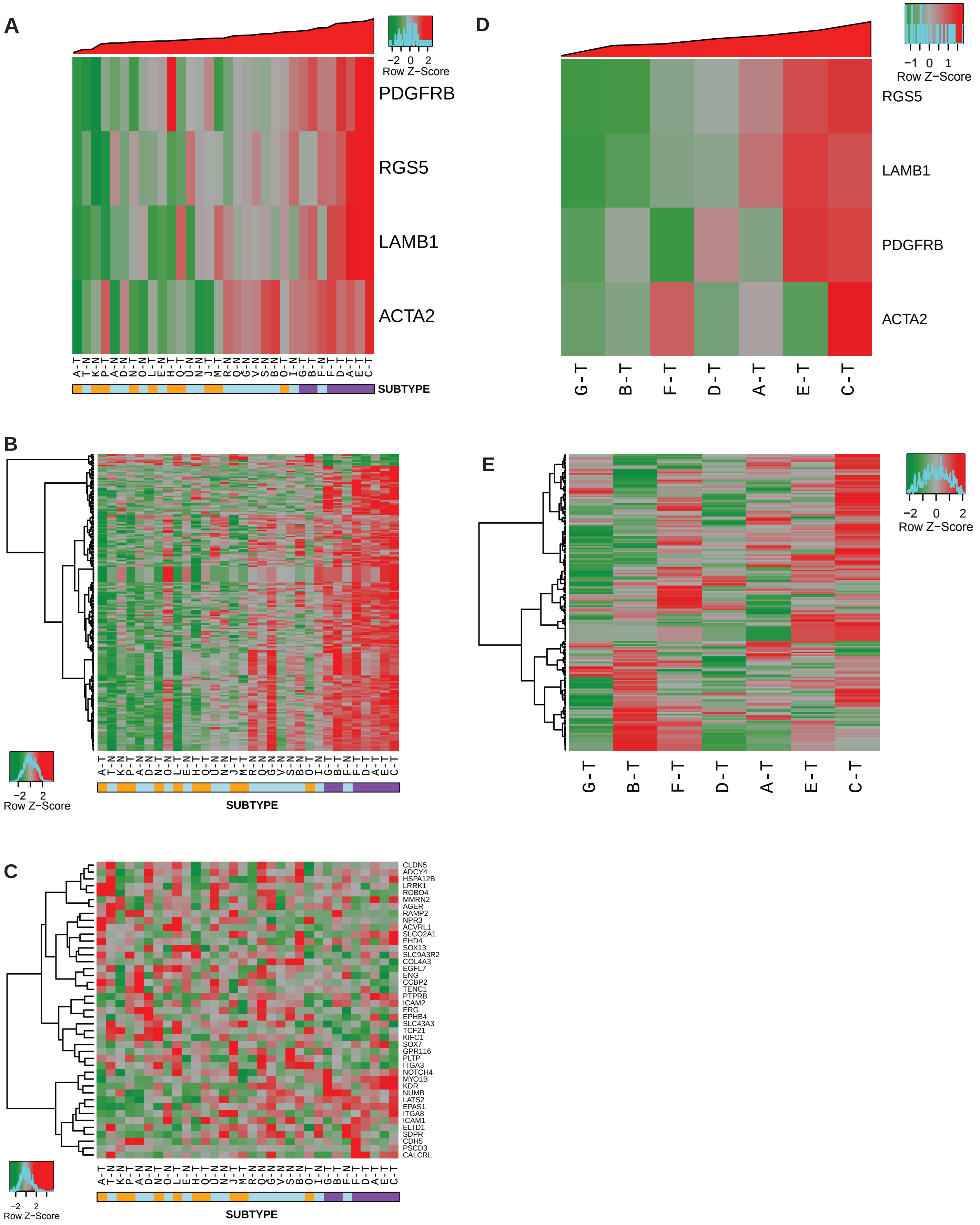

Supplement: Additional file 2 — Supplementary Figures S1 through S6. Supplementary Figure S1 Specific enrichment of the vasculature by laser capture microdissection. Supplementary Figure S2 Heatmap depicting differential gene expression after class discovery within the set of all microdissected endothelial samples and principal component analysis (PCA) of tumor endothelial samples. Supplementary Figure S3 Characteristics of the studies in which other tumor vascular signatures were generated, heatmaps representing expression of antiangiogenic genes and the pericyte marker PDGFRβ in tumor vasculature, and heatmaps of clustering induced by other tumor vascular signatures in the current dataset. Supplementary Figure S4 Images of anti-ACTA2 IHC, as well as of anti-ACTA2/LAMB1 and anti-PECAM1 IHC carried out on consecutive sections. Supplementary Figure S5 qRT-PCR validation for selected genes identified from analyses of microarray data. Supplementary Figure S6 Analyses of correlation between cell-type marker content and expression of genes differentially expressed between the A and B subtypes. [file bcr3246-S2.PDF]
