# Supplementary material for: Gene-expression profiling of microdissected breast cancer microvasculature identifies distinct tumor vascular subtypes
Source: Breast Cancer Res. 2012 Aug 20;14(4):R120. doi: 10.1186/bcr3246 (PMC3680943; doi:10.1186/bcr3246)
Supplement: Additional file 3 — Supplementary Materials and Methods. Supplementary Materials and Methods with corresponding references. [file bcr3246-S3.DOC]

**Additional File 3 - Supplementary Materials and Methods**

**Immunohistochemistry (IHC) for PECAM1**

To visualize endothelial cells for LCM, frozen tissue sections were fixed with cold (4ºC) methanol for 5 minutes and then subjected to IHC using an antibody directed against PECAM1 prior to laser capture microdissection (LCM). A monoclonal anti-human PECAM1/CD31 antibody (Cat. No. BBA7, R&D Systems, Minneapolis, MN) was applied to sections at a 1:100 dilution (in PBS-T) for 2 minutes. A secondary antibody (peroxidase-conjugated AffiniPure F(ab)_2_ fragment goat anti-mouse IgG, Cat. No. 115-036-062, Jackson ImmunoResearch Laboratories, West Grove, PA) was then applied at a 1:25 dilution (in PBS-T) for 2 minutes. No washing was conducted between incubations with primary and secondary antibodies. Sections were rinsed with PBS-T and then quickly immersed 3-4 times in PBS-T. Excess liquid was removed and DAB+ Liquid (Cat. No. K3467, DAKO Canada, Mississauga, ON, Canada) was applied for 2 minutes. RNase inhibitors (SUPERase In^TM^ RNase Inhibitor (Applied Biosystems, Austin, TX) and RNase-Inhibitor (Roche Diagnostics) were added to primary and secondary antibodies, as well as to the DAB+ Liquid. Subsequently, slides were stained with hematoxylin (Surgipath Canada, Winnipeg, MN, Canada) for 2 seconds, washed by 5-7 rapid immersions in DEPC-treated water, and dehydrated by immersion in 95% ethanol (60 seconds, twice); 100% ethanol (60 seconds, once); xylene (5 minutes, twice). Slides were air-dried for 20 minutes and then subjected to LCM.

**Immunohistochemistry for ACTA2 and LAMB1**

To visualize ACTA2 and LAMB1 localization, immunohistochemistry was performed according to standard methods. Briefly, 5 µm sections from frozen samples were prepared on a cryostat, fixed in cold (-20ºC) acetone for 10 minutes, and air-dried. Endogenous peroxidases were blocked with blocking solution (Dako, K4006), and non-specific binding was blocked by incubation with 10% (LAMB1) or 5% (ACTA2) normal goat serum. Incubation with primary antibodies was performed as follows: anti-ACTA2 antibody (Sigma, A2547) at 1:10,000 for 30 min at RT or anti-LAMB1 (Millipore, MAB 1928) at 1:300 overnight at 4ºC. Sections were rinsed with TBS containing 0.1% Tween-20 and incubated with labeled polymer-HRP anti-mouse (Dako, K4006) (ACTA2) or goat anti-rat IgG (Jackson ImmunoResearch Labs, 112-035-167) at 1:250 (LAMB1) for 30 min at RT. Standard diaminobenzidine (DAB) staining was used to visualize immunoreactivity. The color development step was carried out for 10 (LAMB1) or 2 (ACTA2) minutes. Sections were then counterstained with Gill II Hematoxylin (Surgipath, 01520), dehydrated and mounted. Specificity of immunostaining was confirmed by carrying out the same protocol with the omission of primary antibodies.

**Quantitation of IHC labeling**

Slides prepared as described above were scanned using an Aperio XT slide scanner (Aperio Technologies, Inc, Vista, CA) at 20x magnification. After manual exclusion of regions containing large vessels or adipose tissue, positive signal in the remaining tissue regions on the acquired images were analyzed using the Aperio Positive Pixel Count algorithm. The resulting absolute counts of positive pixels for each marker (ACTA2 or LAMB1) were then corrected for PECAM1 staining as quantified in the same manner on an adjacent serial section (Fig. S3). Significance was assessed by standard t-test.

**Gene expression analysis**

Microarray data were extracted from images of scanned slides using Feature Extraction Software (v. 7.5.5.1, Agilent Technologies, Mississauga, ON, Canada) set to the default parameters. Outlier features on the arrays were flagged using the same software package. Data preprocessing and normalization was automated using the BIAS system [1]. Raw feature intensities were background corrected using the RMA background correction algorithm [2, 3], and the resulting expression estimates were converted to log2 ratios. Within-array normalization was performed using a spatial Loess followed by an intensity-dependent Loess correction [4]. Median absolute deviation scale normalization was used to normalize between arrays [5]. Principal component analysis (PCA) on tumor endothelial samples was carried out using the prcomp module in R. Further analyses are described in main text.

Technical validation for selected genes was carried out by performing quantitative real-time PCR (qRT-PCR) as described below. Results are depicted in Fig. S5.

**Pathway enrichment analysis**

We calculated overrepresentation of differentially expressed genes against Gene Ontology (GO) annotations [6] using Fisher’s exact test at a significance threshold of P ≤0.05. Comparison against the Kyoto Encyclopedia of Genes and Genomes (KEGG) [7] was performed by combining the p-values for differential expression derived using the unweighted z-transform method for every gene in each pathway [8]. GO and KEGG annotations were accessed from the R/Bioconductor annotation package hgug4112a (v2.0.1). Comparisons against the Molecular Signatures Database (v2.5) were performed using Gene Set Enrichment Analysis (GSEA) [9].

**Quantitative Real-Time PCR (qRT-PCR)**

All qRT-PCR reactions were performed using amplified RNA (aRNA) samples generated from microdissected cells (as used for microarray-based gene expression profiling) as a template. The template aRNA for qRT-PCR was generated in separate reactions from those used for microarray-based profiling. The aRNA concentration was measured using RiboGreen (Invitrogen Canada, Burlington, ON, Canada). 25 ng of aRNA were reverse transcribed in a final volume of 20 μL (1xRT buffer, 20 units of Protector RNase Inhibitor (Roche Diagnostics Canada, Laval, QC, Canada), 10 units of Transcriptor reverse transcriptase (Roche Diagnostics Canada), 1mM dNTPs and 10μM random nonamer primers). Samples were incubated at 25°C, 10 minutes; 55°C, 30 minutes; 85°C, 5 minutes.

All primers were designed using OligoPerfect™ Designer software (Invitrogen) to map within 300 nucleotides of the 3’-end of the appropriate mRNA. Primer sequences were:

**KRT8**

forward, CTGGGAAGGAGGCCGCTATG;

reverse, TAGCTGGAGGCATGGGCAAG

**PECAM1**

forward, TGGGAGAGAGGCTGCTGTCA

reverse, GAGGGTCCCTGCAGCTGTGT

**PDGFRβ**

forward, GAAGGGGGTGCAGGAAGCTC

reverse, CCCACTCTCCCTCCTTGGAC

**TLR2**

forward, TGGGTAAATCTGAGAGCTGCGATA

reverse, CAGCACCCCAGACAAAATTTGA

**FADD**

forward, TGTGAGGATTATGGGTCCTGCAA

reverse, TACGAGATCCCGCTGCCTTG

**SFRP2**

forward, CATGTATCACATTCCAGCTACA

reverse, GCTACAATGGGTTTAATTTGC

**LAMB1**

forward, GGAAGGAGAAGTCCGTTCACCTCC

reverse, CCTTGTTCACCTCAGCCATTTT

**ANGPT2**

forward, CGCTGCTGTCACAACCAAGA

reverse, TGCTCAGAAGAATGCAGTTCCA

**MET**

forward, TCGGGGAAACATCCCATCAA

reverse, CAGCTGCAGGTATAGGCAGTGACA

**LVVE1**

forward, GCACATAGTAGAACGCTATCTGGGAAG

reverse, GCATCTCTCAGTCCCTTCTTGTTGG

**SPARC**

forward, TGGAGTTGGTGAATCGGTTG

reverse, CAGCCTGTGAGATCCGACCA

**TFF3**

forward, CCCCTGCAGGAAGCAGAATG

reverse, CGGGAGCAAAGGGACAGAAA

**GNG10**

forward, CATTAGGCTGCCCCATCGTG

reverse, TCATTTACAATGTTCTGTGGGTCACAT

**FAM8A1**

forward, TGGGAATAAAGGGCTTTTTGAGG

reverse, CCAGGCAAACATGGGATGTC

Samples used for qRT-PCR were prepared in a final volume of 20 μl (containing 1x LightCycler® FastStart DNA Master SYBR Green I (Roche Diagnostics), 0.5 µmol/L of each primer, and 1 μl input cDNA (equivalent to 1.25 ng aRNA)). Standard PCR parameters were used according to the manufacturer's instructions. Briefly, these consisted of: 10 minutes pre-incubation at 95°C, 45 cycles of denaturation at 95°C for 10 seconds, annealing at 5°C below the lower calculated primer melting temperature for 4 seconds and extension at 72°C for a time equivalent to the amplicon (bp)/25 sec. Reactions were performed using a Roche LightCycler (Roche Diagnostics, Version 1.2) or a Roche LightCycler 480. Crossing points (CPs) were determined from the second derivative maximum on the PCR amplification curve and automatically calculated using the LightCycler 3.5 software (Roche Diagnostics). A melt curve analysis was conducted for each reaction after the final cycle to assure that a single product was amplified. Agarose gel electrophoresis confirmed that the single PCR product was of the predicted length.

Dilution series of cDNA (equivalent to aRNA concentrations of 50ng/ul, 10ng/ul, 5ng/ul, 2.5ng/ul, 1ng/ul and 0.5ng/ul) generated from amplified aRNA derived from Universal Human Reference RNA (Stratagene, La Jolla, CA) plus HUVEC cells (kindly provided by Dr. Sabbah Hussain) were used to generate standard curves of CP vs. log (input RNA amount) for reactions for PECAM1 and KRT8; for all other reactions, cDNA for the dilution series was generated from pooled aRNA to ensure similar amplification efficiencies. The CP values of the unknown samples were transformed to concentration values using these standard curves.

**SUPPLEMENTARY REFERENCES**

1. Finak G, Godin N, Hallett M, Pepin F, Rajabi Z, Srivastava V, Tang Z: **BIAS: Bioinformatics Integrated Application Software.** *Bioinformatics* 2005, **21**:1745-1746.

2. Irizarry RA, Bolstad BM, Collin F, Cope LM, Hobbs B, Speed TP: **Summaries of Affymetrix GeneChip probe level data**. *Nucleic acids research* 2003, **31**:e15.

3. Irizarry RA, Hobbs B, Collin F, Barclay YDB, Antonellis KJ, Scherf U, Speed TP: **Exploration, normalization, and summaries of high density oligonucleotide array probe level data**. *Biostatistics* 2003, **4**:249-264.

4. Smyth GK, Speed T: **Normalization of cDNA microarray data.** *Methods* 2003, **31**:265-273.

5. Yang Y, Buckley M, Speed T: **Analysis of cDNA microarray images.** *Brief Bioinform* 2001, **2**:341-349.

6. Ashburner M, Ball CA, Blake JA, Botstein D, Butler H, Cherry JM, Davis AP, Dolinski K, Dwight SS, Eppig JT, Harris MA, Hill DP, Issel-Tarver L, Kasarskis A, Lewis S, Matese JC, Richardson JE, Ringwald M, Rubin GM, Sherlock G: **Gene ontology: tool for the unification of biology. The Gene Ontology Consortium.** *Nat Genet* 2000, **25**:25-29.

7. Kanehisa M, Goto S: **KEGG: kyoto encyclopedia of genes and genomes**. *Nucleic Acids Res* 2000, **28**:27--30.

8. Whitlock MC: **Combining probability from independent tests: the weighted Z-method is superior to Fisher's approach.** *J Evol Biol* 2005, **18**:1368-1373.

9. Subramanian A, Tamayo P, Mootha VK, Mukherjee S, Ebert BL, Gillette MA, Paulovich A, Pomeroy SL, Golub TR, Lander ES, Mesirov JP: **Gene set enrichment analysis: a knowledge-based approach for interpreting genome-wide expression profiles**. *Proc Natl Acad Sci U S A* 2005, **102**:15545--15550.
